# Neuroprotective effects of naltrexone in a mouse model of post-traumatic seizures

**DOI:** 10.1038/s41598-024-63942-8

**Published:** 2024-06-12

**Authors:** Saul Rodriguez, Shaunik Sharma, Grant Tiarks, Zeru Peterson, Kyle Jackson, Daniel Thedens, Angela Wong, David Keffala-Gerhard, Vinit B. Mahajan, Polly J. Ferguson, Elizabeth A. Newell, Joseph Glykys, Thomas Nickl-Jockschat, Alexander G. Bassuk

**Affiliations:** 1 Stead Family Department of Pediatrics , Carver College of Medicine, University of Iowa, 25 South Grand Ave, 2040 MedLabs, Iowa City, IA 52242 USA; 2 Department of Psychiatry, University of Iowa, Iowa City, IA USA; 3 Department of Radiology, University of Iowa, Iowa City, IA USA; 4 Iowa Neuroscience Institute, University of Iowa, Iowa City, IA USA; 5 Department of Neurology, University of Iowa, Iowa City, IA USA; 6 Department of Psychiatry and Psychotherapy, Otto-von-Guericke University, Magdeburg, Germany German Center for Mental Health (DZPG), partner site Halle-Jena-Magdeburg, Germany Center for Intervention and Research on adaptive and maladaptive brain Circuits underlying mental health (C-I-R-C), Halle-Jena-Magdeburg, Germany; 7 Department of Ophthalmology, Stanford University, Palo Alto, CA USA

**Keywords:** Mu-opioid receptors, Naltrexone, Traumatic brain injury, Nitro-oxidative stress, Neuroinflammation, Neurodegeneration post-traumatic seizures, Epilepsy, Inflammation, Brain injuries

## Abstract

Traumatic Brain Injury (TBI) induces neuroinflammatory response that can initiate epileptogenesis, which develops into epilepsy. Recently, we identified anti-convulsive effects of naltrexone, a mu-opioid receptor (MOR) antagonist, used to treat drug addiction. While blocking opioid receptors can reduce inflammation, it is unclear if post-TBI seizures can be prevented by blocking MORs. Here, we tested if naltrexone prevents neuroinflammation and/or seizures post-TBI. TBI was induced by a modified Marmarou Weight-Drop (WD) method on 4-week-old C57BL/6J male mice. Mice were placed in two groups: non-telemetry assessing the acute effects or in telemetry monitoring for interictal events and spontaneous seizures both following TBI and naltrexone. Molecular, histological and neuroimaging techniques were used to evaluate neuroinflammation, neurodegeneration and fiber track integrity at 8 days and 3 months post-TBI. Peripheral immune responses were assessed through serum chemokine/cytokine measurements. Our results show an increase in MOR expression, nitro-oxidative stress, mRNA expression of inflammatory cytokines, microgliosis, neurodegeneration, and white matter damage in the neocortex of TBI mice. Video-EEG revealed increased interictal events in TBI mice, with 71% mice developing post-traumatic seizures (PTS). Naltrexone treatment ameliorated neuroinflammation, neurodegeneration, reduced interictal events and prevented seizures in all TBI mice, which makes naltrexone a promising candidate against PTS, TBI-associated neuroinflammation and epileptogenesis in a WD model of TBI.

## Introduction

Traumatic brain injuries trigger focal and diffuse injury response, increasing the probability of developing post-traumatic seizures (PTS) and post-traumatic epilepsy (PTE)^1–4^. If PTS is debilitating enough, it can develop into PTE, the most common form of acquired epilepsy, accounting for ~20% of symptomatic epilepsies^2,5^. PTE is a complex, broadly heterogeneous condition, and its pathogenic mechanisms in the context of TBI and other traumas are incompletely understood. A neuroinflammatory response immediately follows traumatic brain injury (TBI) and is mediated by cytokines, chemokines, and free radicals which can promote PTS/PTE^6–8^. TBI also promotes blood–brain barrier breakdown, further exacerbating inflammation^9,10^.

Opioid receptors, particularly mu-opioid receptors (MORs), regulate neuroinflammatory responses and cause inflammation when overstimulated. Studies have shown that the MOR agonist morphine activates the transcription factor NF-ĸB, which in turn mediates a substantial immune response in microglia, resulting in inflammation. In contrast, silencing MORs through siRNA reduces NF-ĸB activation and impairs glial activation and transcription of inflammatory genes^11^. Overstimulation of MORs also drives redox signaling and facilitates activation of various transcription factors in microglia, astrocytes, and neurons, at least in part via mitogen-activated protein kinase (MAPK) signaling^12^. In glial cells, the signaling crosstalk between MOR and MAPK enhances the release of inflammatory cytokines (e.g., TNFα, IL-1β, and IL-6) and of inducible nitric oxide synthase (iNOS), which jointly increases the oxidative load, damaging cellular components^13,14^. Conversely, antagonizing or deleting MORs decreases neuroinflammation by attenuating oxidative stress and reducing inflammatory cytokines. These findings indicate that MOR signaling contributes to inflammation^15–17^.

Opioid receptors not only modulate neuroinflammation but also play a complex role in seizures and can generate either a pro- or anti-convulsive response depending on the dose, the drug administered, and the opioid receptor activated. There have been a number of studies demonstrating a positive relationship between MOR and development of epilepsy in models such as amygdaloid kindling and temporal lobe epilepsy^18–20^. We recently reported that naltrexone, a MOR antagonist, decreases seizure-like activity in zebrafish and mice in vitro and in vivo^21^. Nonetheless, studies evaluating naltrexone’s long-term anti-epileptic properties are still lacking. Here, we investigated the anti-inflammatory and anti-convulsive role of naltrexone in a modified Marmarou weight-drop (WD) model of TBI. We hypothesize that an increase in MOR activity in the neocortex after TBI would drive neuroinflammation and neurodegeneration, promoting epileptogenesis. In contrast, we expect that antagonizing MOR soon after TBI should reduce the severity of these insults and prevent the development of seizures. Therefore, we evaluated TBI-induced damage and the neuroprotective effects of naltrexone in the acute and chronic phases following TBI. The onset and progression of seizures was tracked by integrated remote video-EEG (vEEG) and by profiling biomarkers which are known to play an important role in the development of epilepsy. These biomarkers include nitro-oxidative stress (iNOS, 3-NT), pro-inflammatory genes (IL-1β, TNFα, IL-12, IFNγ), microgliosis, neurodegeneration and cytokines^22–24^. We found an increase in some of those biomarkers in the neocortex of TBI mice, whereas intervention with naltrexone after injury decreased them.

In the context of post-traumatic epilepsy, damage to white matter can play a significant role in the development and manifestation of seizures. The disruption of neural pathways and communication between brain regions due to white matter injury may contribute to the abnormal electrical activity characteristic of epilepsy. Additionally, white matter damage can result in gliosis, a process where glial cells proliferate in response to injury, which further alters the brain's functioning and increases the likelihood of epileptic seizures^25^. Reports have shown that substantial number of people who have developed epilepsy after moderate to severe traumatic brain injury have non-lesional MRI, meaning that the injury was a diffuse injury and the axonal fibers are sensitive to the shearing and stretching forces that occur after TBI^26–28^. In our model, we observed similar damage to the white matter after TBI, whereas naltrexone prevented widespread injury to the white matter as determined through diffusion tensor magnetic resonance imaging. Overall, understanding the interplay between post-traumatic epilepsy and seizures and white matter damage is crucial for developing effective treatments and interventions to manage seizures and improve outcomes for individuals with TBI and PTS/PTE. Our results indicate that targeting MORs can provide a therapeutic advantage against neuroinflammation, which can start immediately after TBI, ultimately preventing the progression of seizures.

## Results

### Naltrexone decreased phosphorylation and expression of MOR in the neocortex

To test whether injury to the brain after TBI alters MOR expression and whether antagonizing MOR after injury normalizes its expression, we started naltrexone treatment two hours after PTZ with additional naltrexone boluses for six days. The pro-convulsant PTZ is a GABA_A_ receptor antagonist used in various rodent models to chemically induce acute seizures. The mice were administered a sub-convulsive dose of PTZ which by itself is unable to drive the inflammatory response seen following a TBI. Rather, it is a useful test to determine seizure susceptibility in mice following the injury, which have been described previously^29–33^. We first evaluated MOR expression and its activation status after TBI. MOR phosphorylation at specific amino acid residue sites is key for its signaling and regulation^34,35^. To elucidate the relationship between MOR phosphorylation and receptor activation, we investigated the phosphorylation status of S375, as this is one of the primary amino acid residues in MOR that is phosphorylated in response to an external stimulus.

**Figure 1:**
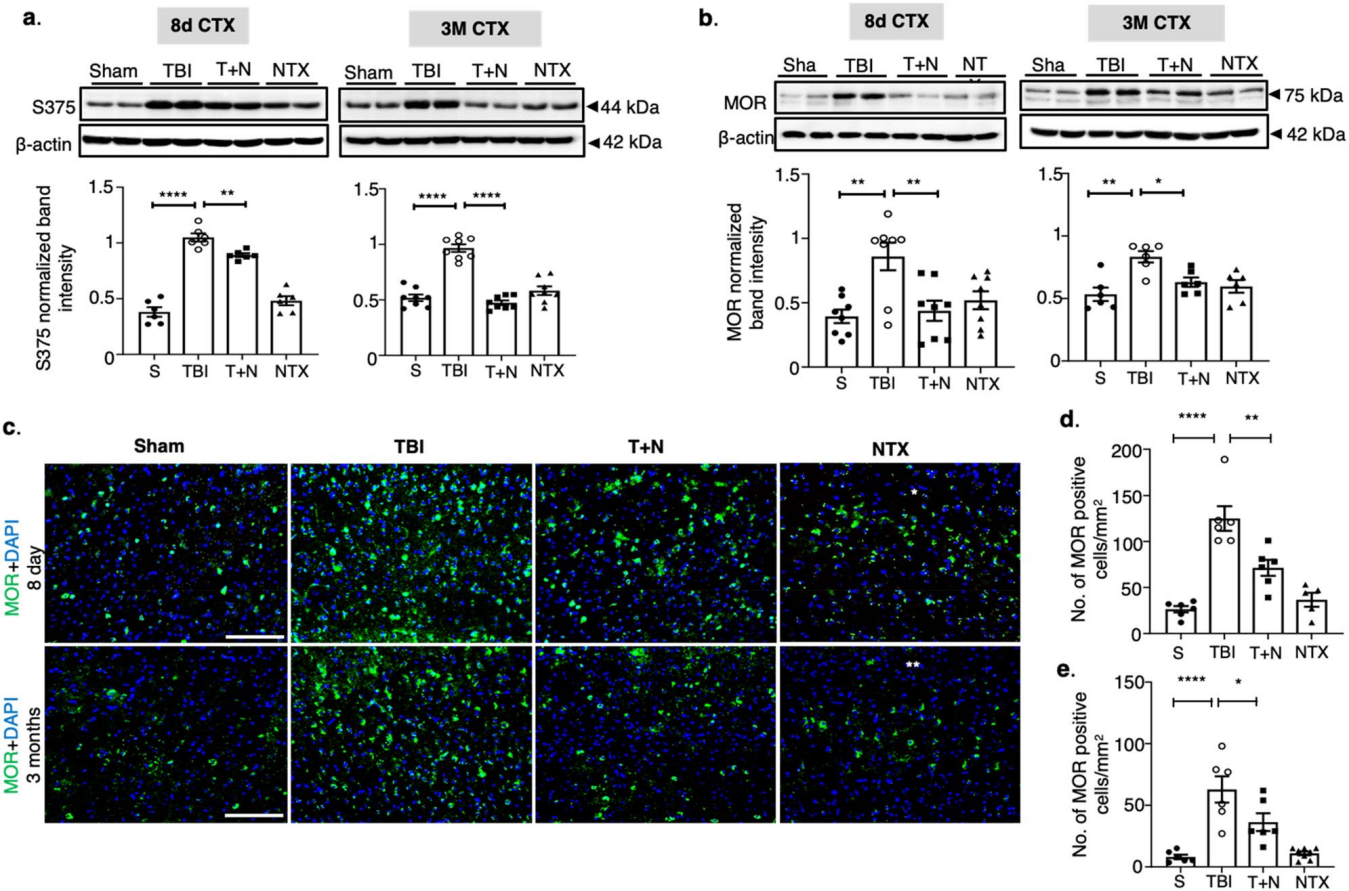
Naltrexone normalized MOR signaling in cortex following TBI. **(a,b)** Western blot analysis of phospho-MOR (S375) and MOR in the CTX tissue extract at 8 days and 3 months post-TBI. TBI increased both S375 phosphorylation (panel 1A) and MOR expression (1b) in the CTX at acute and chronic time-points compared to sham, and NTX treatment (T + N group) significantly reduced the effect of TBI. **(c)** MOR immunostaining in the CTX. Green represents MOR, and blue is DAPI **(d,e)**. Quantification of MOR-positive cells (with DAPI) in the CTX at **(d)** 8 days and **(e)** 3 months post-TBI, both illustrating reduction of MOR-positive cells with NTX treatment. All groups were compared to each other using one-way ANOVA with Tukey’s post-hoc test; *p < 0.05, **p < 0.01, ****p < 0.0001; *n* = 6–8 (3–4 sections per animal). All the data is represented as standard error mean (SEM). Scale: all 100 μm. *S* sham, *TBI* traumatic brain injury, *T + N* TBI with naltrexone, *NTX* naltrexone only, without TBI, neocortex (CTX).

We observed a significant increase in phospho-MOR in the neocortex (CTX) at both 8 days (****p =  < 0.0001, sham vs. TBI; *p = 0.024, TBI vs. T + N) and 3- months post-TBI (****p =  < 0.0001, sham vs. TBI; ****p < 0.0001, TBI vs. T + N), when compared to sham mice. Naltrexone treatment significantly decreased phospho-MOR levels at both time-points, whereas naltrexone on its own had no effect (Fig. 1a). To further examine whether S375 phosphorylation has any effect on MOR expression, we measured MOR total protein levels. Interestingly, both MOR protein levels and S375 phosphorylation had similar trends. In the neocortex of TBI mice, MOR levels were significantly higher compared to sham at acute and chronic time points. Naltrexone treatment after TBI (T + N group) substantially reduced MOR protein levels. In the absence of TBI, naltrexone had no effect (Fig. 1b). IHC assay also confirmed an increase in MOR-positive cells in the neocortex of TBI mice. In contrast, significantly less MOR immunopositive cells were observed in naltrexone-treated mice (T + N) at both time points (Fig. 1c–e). Our western blot and IHC analyses confirmed that TBI increased the expression of MOR in the neocortex and naltrexone treatment, after TBI, reduced MOR expression.

### Naltrexone reduced TBI-induced p38 phosphorylation and nitro-oxidative stress

MOR can activate MAPK, a pivotal regulator of inflammatory gene transcription and activation^12,36^. MAPK pathways respond to various stimuli and transduce various intra- and extracellular signals, such as stress. We hypothesized that TBI-induced stimulation of MOR triggers MAPK signaling via p38 phosphorylation, which enhances nitro-oxidative stress and promotes neuroinflammation. Immunoblots of neocortex tissue lysates showed significant upregulation of phosphorylated p38 MAPK at 8 days and 3 months post-injury.

**Figure 2:**
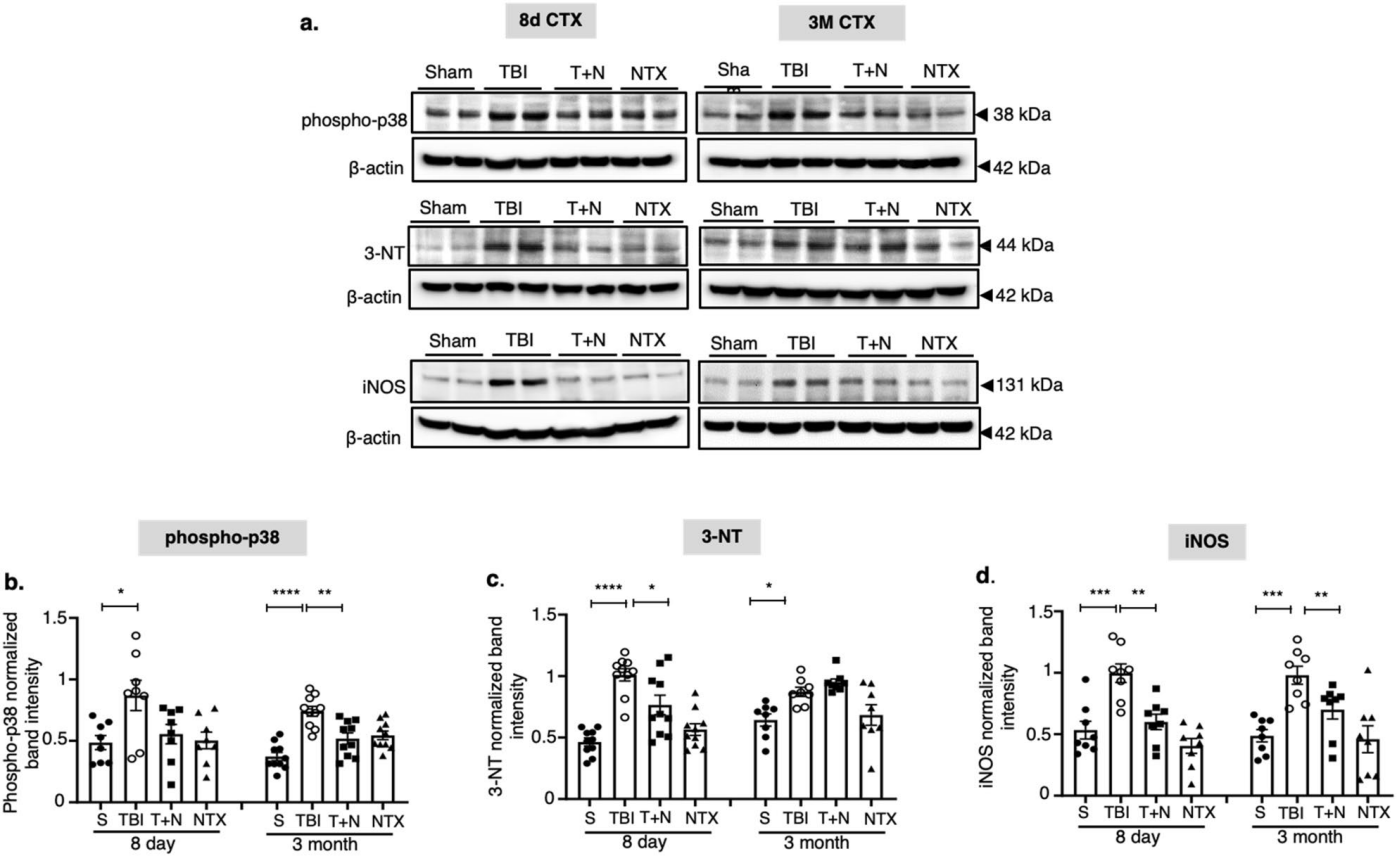
Phospho-MAPK levels and nitro-oxidative stress markers in the CTX. Western blot analysis of phospho-p38 (MAP kinase), 3-NT (a marker of protein nitrosylation), and iNOS from the CTX at 8 days and 3 months post-TBI **(a)**. Increased levels of phospho-p38, 3-NT and iNOS were detected in the CTX at both time points compared to sham **(b–d)**. NTX mitigated all biomarker levels when compared to the TBI group (except 3-NT at 3 months). *p < 0.05, **p < 0.01, ***p < 0.001, ****p < 0.0001; One-way ANOVA with Tukey’s post hoc test; *n* = 6–8. All the data is represented as standard error mean (SEM). *S* sham, *TBI* traumatic brain injury, *T + N* TBI with naltrexone, *NTX* naltrexone only, without TBI.

Naltrexone, after TBI, reduced the levels of p38 MAPK phosphorylation at both time points (Fig. 2a,b). p38 MAPK phosphorylation activation correlated with phosphorylation of S375 providing evidence of MOR activation. Two markers of nitro-oxidative stress, 3-nitrotyrosine (3-NT) and iNOS, were also substantially elevated in the neocortex of TBI mice, and elevated levels persisted for at least 3 months post-injury. Naltrexone (T + N) reduced iNOS at both time points, but 3-NT levels were only reduced at 8 days and not at 3 months (Fig. 2c,d). Thus, naltrexone has both early and partial long-term neuroprotective effects in mitigating TBI-induced nitro-oxidative stress in the neocortex.

### Naltrexone decreased TBI-induced expression of inflammatory cytokine genes

MAPK phosphorylation of p38, and the subsequent stimulation and nuclear translocation of NF-kB, regulate the transcription of inflammatory genes^37,38^. Since we observed increased phosphorylation of phospho-p38 in the neocortex, we investigated whether the expression of inflammatory cytokines in the neocortex increases subsequent to the MOR-dependent phosphorylation of MAPK that is induced by TBI. We performed quantitative RT-PCR to determine mRNA expression of various cytokines in the neocortex.

**Figure 3:**
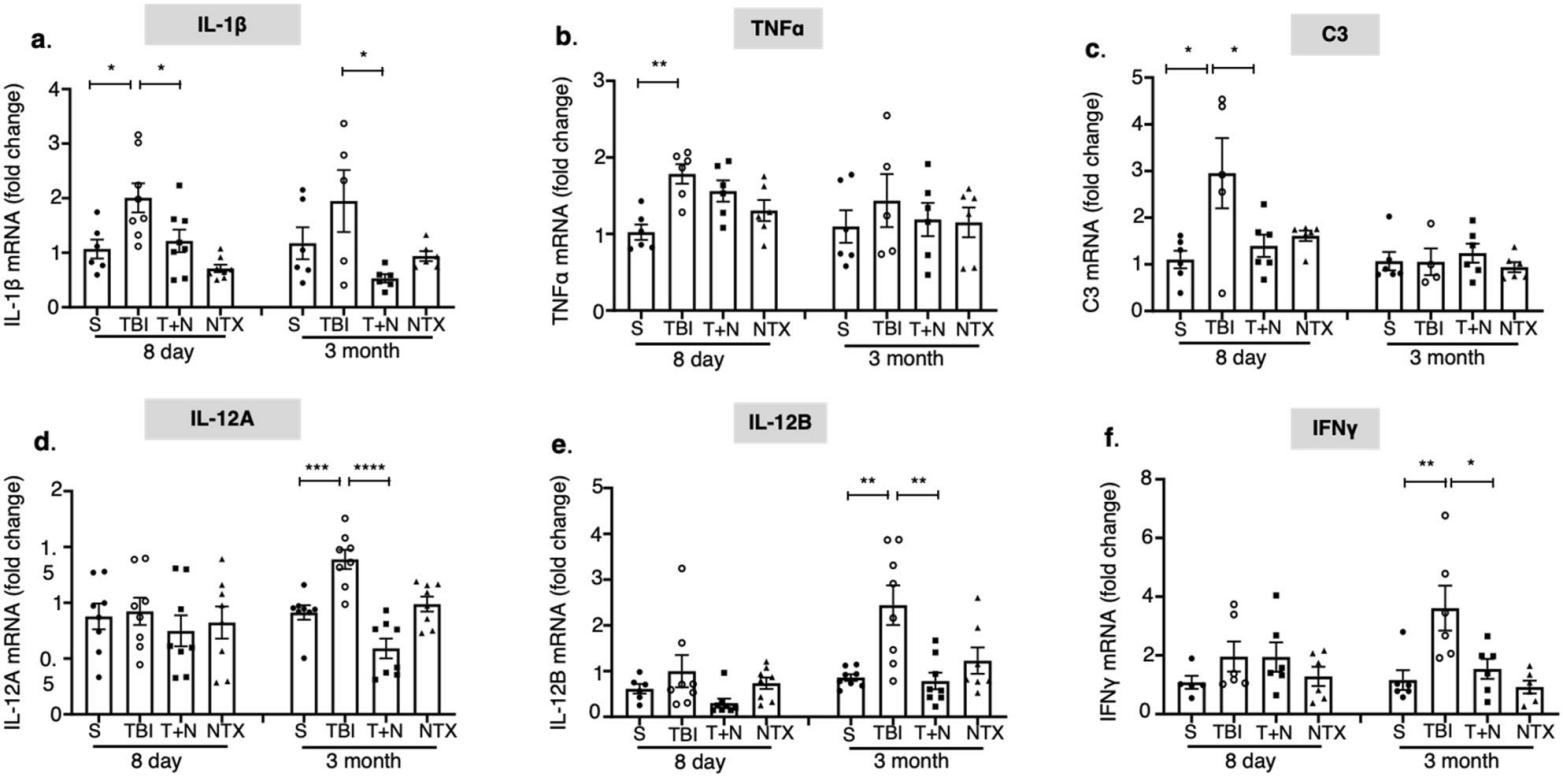
mRNA expression of inflammatory cytokines. mRNA levels were measured by qRT-PCR. *IL-1β*, *TNFα*, and complement protein *C3* (encode inflammatory cytokines) were significantly higher at 8 days **(a–c)**, whereas *IL-12A*, *IL-12B* and *IFNγ* mRNAs were increased at 3 months post-injury **(d–f)**. NTX treatment attenuated *IL-1β* and *C3* expression at 8 days, and decreased *IL-12A* and *IL-12B* at both time points **(d,e)**, whereas *IFNγ* mRNA levels **(f)** were decreased at 3 months after TBI. *p < 0.05, **p < 0.01; One-way ANOVA with Tukey’s post hoc test; *n* = 5–8. All the data is represented as standard error mean (SEM). *S* sham, *TBI* traumatic brain injury, *T + N* TBI with naltrexone, *NTX* naltrexone only, without TBI.

In the TBI group compared to sham, we detected a significant increase in *IL-*1β, *TNFα*, and complementary protein *C3* transcripts and no changes in *IL-12A-B* and *IFNγ* expression at 8 days. At 3 months, mRNA expression of *IL-12A*, *IL-12B*, and *IFNγ* was significantly elevated post-TBI while expression of the other markers was close to the basal levels (Fig. 3). Naltrexone treatment after TBI reduced the expression of the inflammatory genes both acutely (IL-1β, C3) and chronically (IL-12A, IL-12B, IFNγ) with no changes to TNFα at either time point.

### Naltrexone mitigated TBI-induced microgliosis and neurodegeneration

As the primary endogenous immune cell in the CNS, microglia are major contributors to the neuroinflammatory response following brain injury. In microglial cells, MOR stimulation promotes an inflammatory phenotype by increasing the expression of inflammatory genes and inducing neurodegeneration through oxidative stress^11,17,39^. To assess the impact of MOR signaling on microglial numbers and neurodegeneration, we performed immunostaining for the microglial marker, IBA1, and for neuronal degeneration/stress marker with fluorescent dye Fluoro-Jade B (FJB) and with neuronal nuclear protein (NeuN) which is a neuronal marker. IBA1 expression correlates with microglia, while FJB is a well-established marker of neuronal degeneration^40–44^.

**Figure 4:**
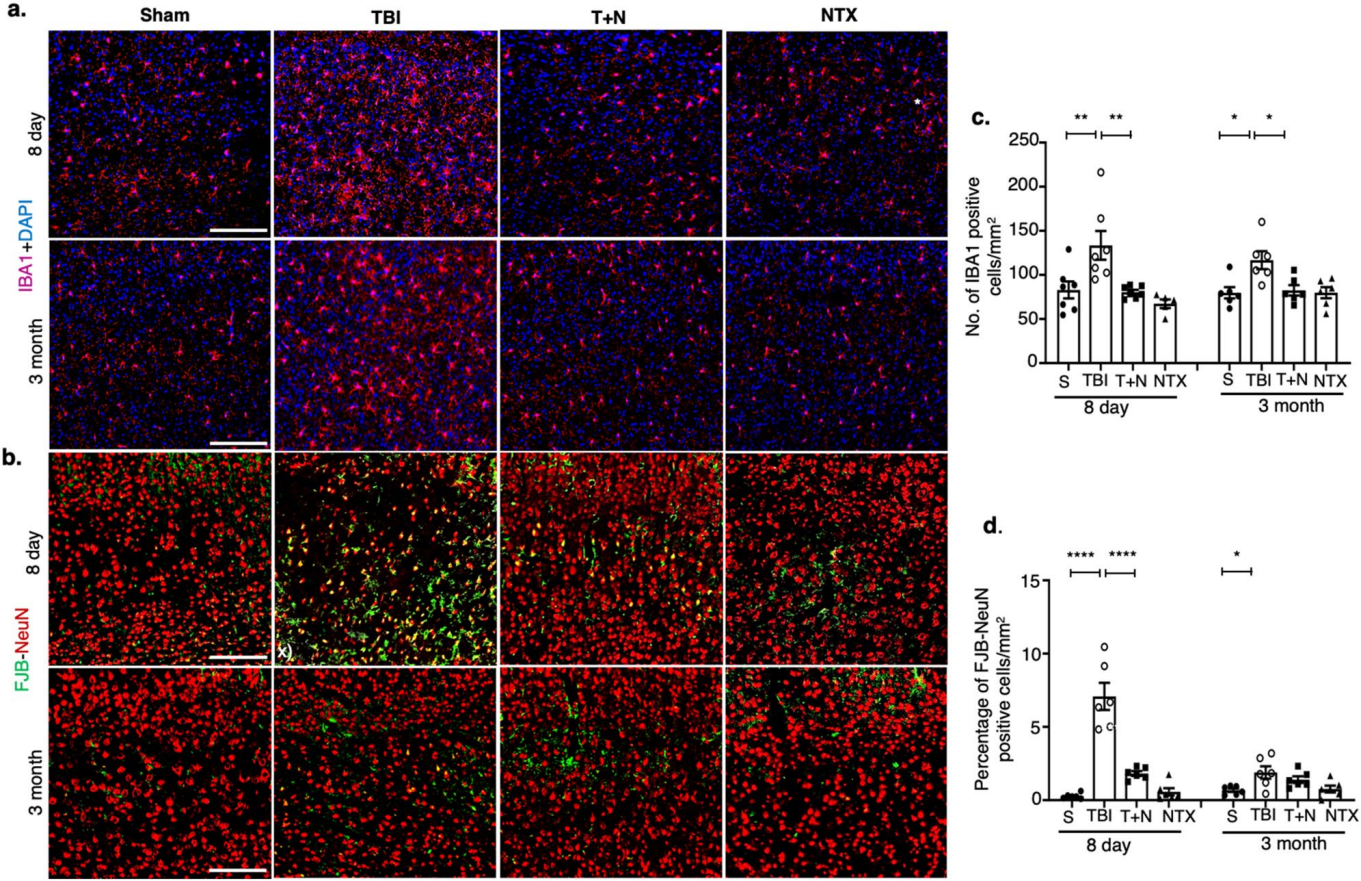
TBI-induced microgliosis and neurodegeneration in the neocortex. (**a**) The total number of IBA1-positive cells were used to quantify microglia. IBA1 (pink) and DAPI (blue) immunostaining in panels in the CTX. Higher number of microglia cells were observed in TBI mice, which were significantly reduced after naltrexone treatment. **(b)** FJB-NeuN (green–red) in the cortex. Yellow staining represents co-localized cells showing degenerated or stressed neurons. **(c)** Count of IBA1-positive cells and quantification of FJB-positive cells **(d)** at 8 days and 3 months after TBI. *p < 0.05, **p < 0.01, ****p < 0.0001, One-way ANOVA with Tukey's post-hoc test; *n* = 6–8 (3 sections per animal). Data is represented as SEM. Scale, all 100 μm. Abbreviations: NTX, naltrexone treatment only, without TBI; T + N, naltrexone treatment, with TBI. *S* sham, *TBI* traumatic brain injury, *T + N* TBI with naltrexone, *NTX* naltrexone only, without TBI, *FJB* fluoro-jade-B, *NeuN* neuronal nuclear protein.

We quantified the total number of IBA1 positive cells. Animals at 8 days and 3 months post-TBI showed acute and chronic increased microglia in the neocortex. Co-labeling for IBA1 and DAPI revealed a substantial increase in IBA1-positive cells at both early and late time points post-injury, compared to sham. Naltrexone-treated animals (T + N group) had significantly fewer IBA1-positive cells in the acute and chronic phases (Fig. 4a,c). FJB staining for neurodegeneration identified higher NeuN-FJB colocalization in the TBI group at early and late time points, indicating increased neuronal degeneration. In TBI brains treated with naltrexone, there was a significant reduction in the number of FJB-positive neurons at 8 days but not at 3 months post-TBI (Fig. 4b,d) which correlates with the partial reduction in oxidative stress produced by 3-NT (Fig. 2c). Fewer NeuN-positive neurons were also detected in the cortex of TBI group, especially in the areas surrounding more densely stained FJB-positive cells, and they recovered significantly better with naltrexone treatment (Fig. 4b; Supporting Table 3). These results demonstrate that TBI increases microglia and neurodegeneration, especially during the acute phase of injury, and this can be prevented by blocking MOR with naltrexone.

### Naltrexone decreased TBI-induced elevation of serum cytokines and chemokines

**Figure 5:**
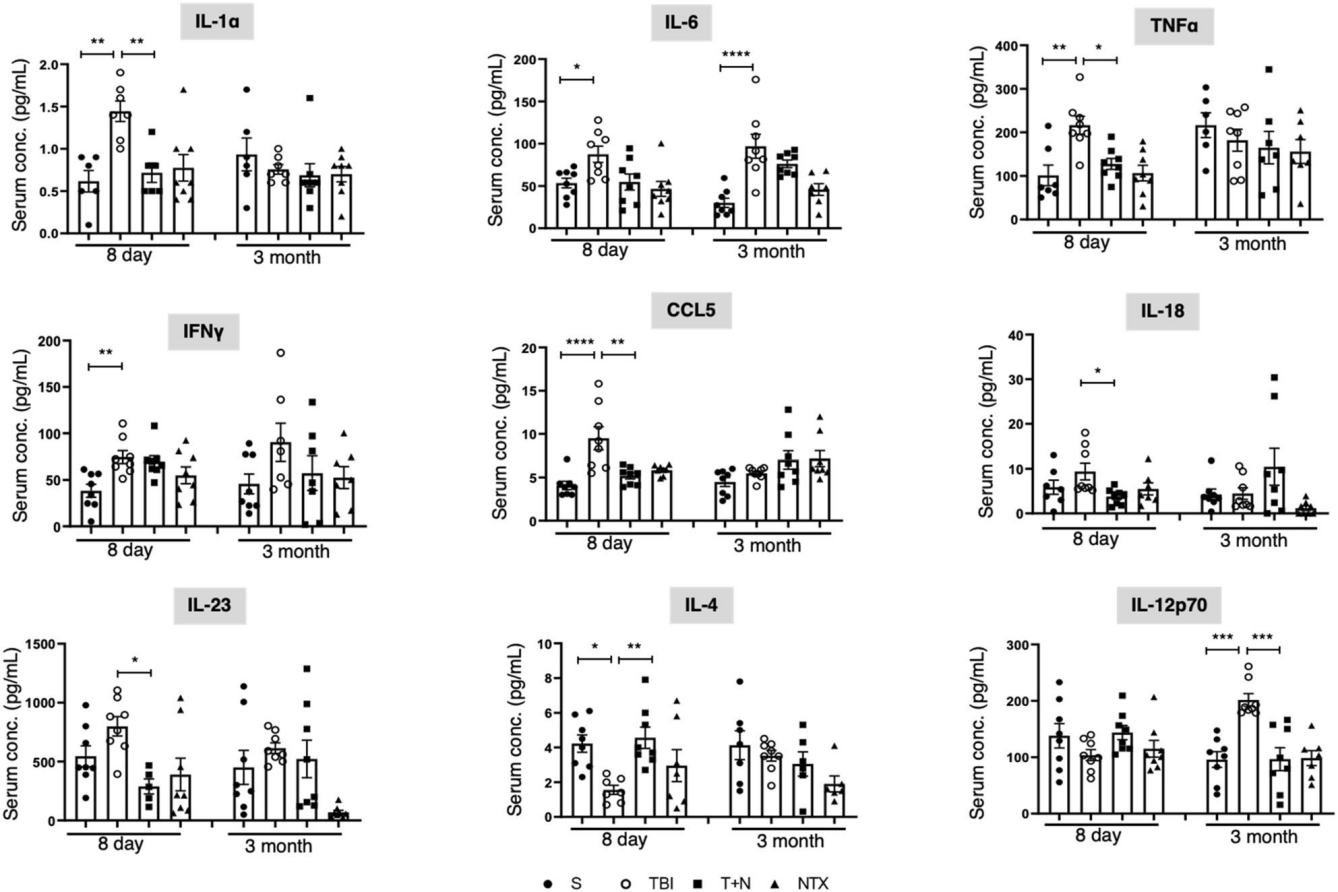
Multiplex cytokine profile for proinflammatory and anti-inflammatory cytokines. The effects of NTX treatment on cytokines and chemokine levels in the serum at 8 days and 3 months post-TBI. Most of the serum cytokine levels were higher in the TBI group at the acute phase, whereas IL-6 and IL-12 were higher during the chronic phase of injury. NTX normalized the serum levels of all the inflammatory cytokines and recovered anti-inflammatory IL-4 levels. *p < 0.05, **p < 0.01, **** < p < 0.001, ****p < 0.0001; One-way ANOVA with Tukey’s post hoc test; *n* = 6–8 per group. Data is represented as SEM. *S* sham, *TBI* traumatic brain injury, *T + N* TBI with naltrexone, *NTX* naltrexone only, without TBI.

To study the systemic effects of TBI, we assessed serum cytokine levels after injury and the impact of naltrexone on cytokines. Serum cytokines were quantified by multiplex ELISA at 8 days and 3 months post-TBI. In the TBI group at 8 days, cytokines IL-1α, IL-6, TNFα, IFNγ, and chemokine CCL5 were significantly elevated, whereas the anti-inflammatory cytokine IL-4 was reduced. Naltrexone, after TBI, (T + N group) effectively reduced levels of IL-1α, IL-6, TNFα, CCL5, IL-18, and IL-23 and restored IL-4 levels to normal at 8 days. At 3 months, only cytokines IL-6 and IL-12p70 were substantially elevated in the serum of injured animals. Of the two inflammatory cytokines, only IL-12p70 levels were significantly decreased by naltrexone (Fig. 5). As for IL-1β and IL-17, cytokines most often elevated in the serum of TBI and human epileptic patients, no changes were observed in their levels when compared to sham at 3 months. In addition, chemokines such as CCL2, CXCL5, CXCL10, growth factor VEGF, and GM-CSF or M-CSF were also unaffected at 3 months in all four groups. A complete list of the cytokines evaluated for all four groups is given in Supporting Table 2.

### Naltrexone treatment of TBI brains protect fiber tract integrity

Fractional anisotropy (FA) is a proxy measure of fiber tract integrity, myelination, and other factors potentially reflecting white matter pathologies in the brain^45^. Decrease in FA is commonly associated with axon loss or myelination abnormalities^46^. Our tract-based spatial statistics analysis showed that TBI was associated with a widespread loss of fiber tract integrity. Following TBI, FA decreased significantly during the acute phase in the long interhemispheric (callosum), peristriatal, thalamic, internal capsule fiber tracts, and other regions of the brain near the injury site, indicating damage or loss of fiber tracts.

**Figure 6:**
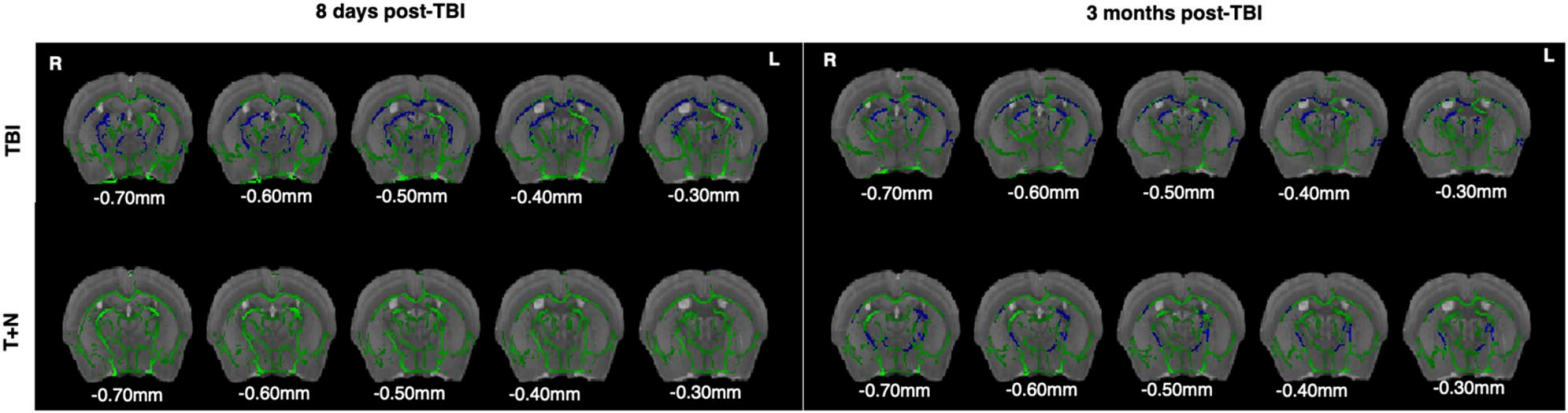
Diffusion tensor imaging measurements of fractional anisotropy of TBI and TBI-naltrexone treated mice. *t-statistical* maps of whole brain diffusion tensor imaging showing differences in FA at 8 days and 3 months post-TBI. Images are overlaid onto the Waxholm template image with the mean FA skeleton shown in green. Statistics were calculated using a randomized algorithm from FMRIB software library (FSL), with 252 possible permutations and threshold-free cluster enhancement (TFCE). The images were thresholded at p < 0.05 (n = 5), with significant decreases in FA shown in blue. Abbreviations: diffusion tensor imaging (DTI); fractional anisotropy (FA); tract-based spatial statistics (TBSS). *TBI* traumatic brain injury, *T + N* TBI with naltrexone.

The effects on FA seen during the acute phase after TBI were also observed in the chronic phase. Strikingly, we detected no FA changes during the acute phase in the naltrexone-treated group. At 3 months, small but significant clusters of FA reduction in long interhemispheric and peristriatal fibers were observed in the naltrexone-treated group, indicating protection of fiber tracts (Fig. 6, Table 1, Supporting Table 4). Therefore, naltrexone’s protective effects during acute and chronic stages might be due to protection against mechanisms such as reducing excitotoxicity, inflammation, and oxidative stress, all of which can damage white matter associated with seizures. It is important to note that long-term treatment with such therapies may have different effects on white matter compared to short-term use. Additionally, adherence to therapies is crucial, as interruptions or changes in medication can impact seizure control and potentially affect white matter integrity. Overall, our data shows that naltrexone prevented changes in FA suggesting that naltrexone protected myelination primarily in regions such as the neocortex, peri hippocampal fiber tracts, corpus callosum, and thalamus.

**Table 1:** Fractional anisotropy (FA) analyses between TBI and TBI/NTX groups showing mean fractional anisotropy values.

Region	CTX		CC		THAL		SC		IC		InC		CP		HF		CBL	
Groups	Pre	Post	Pre	Post	Pre	Post	Pre	Post	Pre	Post	Pre	Post	Pre	Post	Pre	Post	Pre	Post
TBI (8d)	0.259	0.240*	0.420	0.394*	0.347	0.321*	0.317	0.296*	0.359	0.306*	0.453	0.401*	0.296	0.278*	0.258	0.243*	0.291	0.265*
T + N (8d)	0.258	0.257	0.400	0.406	0.359	0.359	0.329	0.303	0.356	0.322	0.441	0.431	0.313	0.324	0.273	0.271	0.302	0.304
TBI (3 M)	0.254	0.234*	0.397	0.369*	0.36	0.326*	0.312	0.280*	0.356	0.283*	0.439	0.393*	0.292	0.296*	0.283	0.271*	0.269	0.242*
T + N (3 M)	0.258	0.225	0.400	0.372	0.359	0.317	0.329	0.251*	0.356	0.273*	0.441	0.384*	0.313	0.286*	0.273	0.252*	0.302	0.253*

Mean FA values of different brain regions of the TBI and T + N groups. All the post-injury values (after TBI) were normalized against the pre-injury (before TBI) group. Therefore, the higher the value, the higher the FA, whereas lower values reflect decreased FA. The asterisk shows the difference in FA values between pre-injury (baseline scans) and post-injury scans.

*CTX* white matter adjacent to neocortex, *CC* corpus callosum, *THAL* thalamic fiber tracts, *SC* fiber tracts adjacent to the superior colliculus, *IC* fiber tracts adjacent to the inferior colliculus, *InC* internal capsule, *CP* white matter adjacent to caudate and putamen, *HF* white matter adjacent to hippocampus, *CBL* cerebellum, *TBI* traumatic brain injury, *T + N* TBI with naltrexone.

### Naltrexone decreased interictal events and prevented post-traumatic seizures

We used video-EEG to record the occurrence of spontaneous seizures and interictal events in mice after TBI and naltrexone treatment. Electrographic seizures were defined as high amplitude frequency discharges lasting for at least 10 s, with spike amplitude three times the baseline, and inter-spike interval of less than 5 s^47–49^. The monitoring of the spontaneous seizures started at least three weeks after TBI.

**Figure 7:**
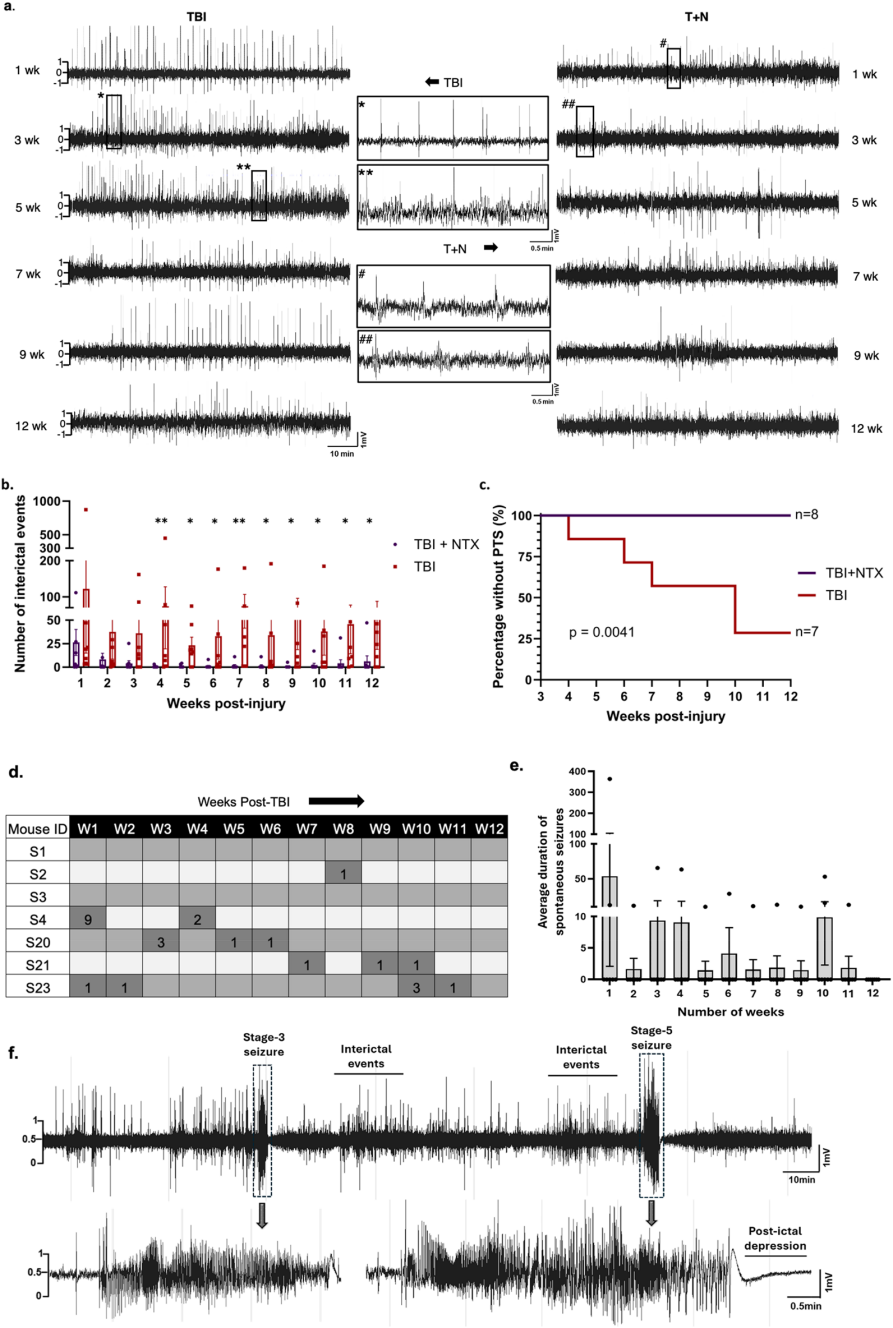
Naltrexone reduced interictal events and prevented post-traumatic seizures. **(a)** Segments of EEG traces showing interictal events between TBI and T + N groups. Asterisks and hashmarks represent the expanded EEG traces from the TBI and T + N groups, respectively.** (b)** The number of interictal events over 3-months was significantly reduced in the T + N group after 4 weeks and onward compared to the TBI group. **(c)** The proportion of mice with seizures following TBI + PTZ is significantly reduced in the T + N group compared to the TBI group. Week-wise representation of the number of spontaneous seizures shown by each mouse that sustained TBI **(d)**, and the average duration of spontaneous seizures **(e)** during the 12 weeks of observation period. Dark grey columns with numbers in figure d represents the number of seizures each mouse (S1–S4; S20–S22) had in that week. The seizure severity stage type, i.e., stage 1–5 for individual mice for each week is illustrated in Supporting Table 5. **(f)** Representative EEG trace showing stage 3 and stage 5 seizure in mice. Stage 3 seizure was smaller in duration with lower spike amplitude, whereas stage 5 seizure was longer and had higher amplitude. Stage 5 seizure also showed post-ictal depression at the end of the seizure. One-way ANOVA Mann–Whitney test *p < 0.05, **p < 0.01, *n* = 7–8. Log rank P-value = 0.0041, n = 7–8 per group, all the data is represented as standard error mean (SEM). *TBI* traumatic brain injury, *T + N* TBI with naltrexone, *PTS* post traumatic seizures.

The automated analysis was performed with MATLAB, and all seizures and interictal events were verified against integrated/synchronized videos associated with the EEG traces on Neuroscore software. Representative EEG traces showing interictal events from TBI and naltrexone-treated mice are illustrated in Fig. 7a. High amplitude single spikes with high frequency were observed in TBI mice, whereas such events were reduced in mice that were treated with naltrexone after injury. Twelve weeks of long-term recording revealed that naltrexone treatment significantly reduced the number of interictal events from the fourth week until the end of the observation period (Fig. 7b). In addition, none of the naltrexone-treated mice showed seizures, whereas 71% of the untreated animals that had TBI showed late post-traumatic seizures (Fig. 7c), revealing naltrexone’s potential anti-seizure role. A seizure due to TBI occurring after first few days of injury were considered as early (within the first week) or late (after first week) post-traumatic seizures^5,50,51^. The number of seizures demonstrated per individual mice, their duration and representative EEG trace of spontaneous seizure is illustrated (Fig. 7d–f). The majority of the mice showed stage 2 (non-convulsive) and stage 5 (convulsive) seizures, according to the modified Racine scale as described^21,47,48,52^. The number and type of seizure is described in Supporting Table 5.

## Discussion

Naltrexone is an FDA-approved opioid antagonist widely used to manage alcohol and opioid dependence^53^. Our previous studies had shown that naltrexone has anti-convulsive properties in zebrafish and mouse models, but did not address if naltrexone had anti-convulsive properties^21^. To address this key translational issue, here we tested naltrexone in a mouse weight drop model of TBI with three months of video-EEG study to assay PTS, and complemented this with an analysis of a series of molecular and biochemical disease biomarkers for inflammation and inflammation-related signaling. Our results show that naltrexone reduced the severity of many of the sequelae of TBI, such as neuroinflammation, nitro-oxidative stress, neurodegeneration, white matter fiber injury, interictal events and notably, PTS. With none of the mice that received NTX post-TBI developing PTS, it provides further evidence that NTX may play a role in modifying the development of PTS.

The effects of naltrexone likely reflect selective effects on the mu opioid class of opioid receptors. Naltrexone is a potent, competitive MOR antagonist with a higher selectivity and affinity for MOR (K_i_ = 1.0 nM) than for delta (DOR; K_i_ = 149 nM) and kappa opioid receptors (KOR; k_i_ = 3.9 nM)^54^. Competitive receptor binding assays showed that the presence of a cyclopropyl methyl group on the nitrogen atom in naltrexone increases its receptor binding affinity for MOR but not KOR or DOR^55,56^. In addition to targeting Asp147 and hydrogen bond interactions at Tyr148, naltrexone also forms additional hydrophobic interactions with Lys233, causing conformational changes in MOR. These interactions cause naltrexone to bind more strongly to MOR than to other opioid receptors, making naltrexone a better and more selective antagonist for MOR^55^.

Ligand binding to MOR causes a sequential and hierarchical multi-site phosphorylation of several amino acid residues leading to the receptor’s fully functional poly-phosphorylated state^57^. For example, morphine stimulates phosphorylation of serine 375 (S375) and threonine 379 (T379) at lower concentrations and T370 and T376 at higher concentrations^58^. In vivo and in vitro proteomic studies on MORs revealed that application of a small amount of agonist (e.g., morphine) enhances phosphorylation of S375, a site phosphorylated early in the MOR-activation response^57^. In light of this, and given that opioid receptors like MOR are known to regulate neuroinflammatory responses, we hypothesized that S375 phosphorylation increases after TBI, initiating MOR receptor activation and signaling^59^. Indeed, we observed increased S375 phosphorylation in the neocortex of injured mice, which correlated well with increased overall MOR expression. Interestingly, the phosphorylation and activation of S375 and MOR persisted even through the chronic phases of injury. The addition of naltrexone demonstrated reduced S375 phosphorylation at both acute and chronic time points, highlighting the possibility of S375 playing a role in MOR expression and signaling after TBI. Nonetheless, more research needs to be done since S375 may be among several residues controlling MOR activation.

MORs activate MAPKs, which in turn regulate diverse cellular functions. Overactivation of MOR can cause cytotoxic oxidative stress by generating excessive reactive oxygen and nitrogen species (ROS/RNS)^11,17,39^. Most of the damage induced by reactive nitrogen species (RNS) is triggered by iNOS and NADPH oxidase (NOX), which generate superoxide anions that combine with nitric oxide to cause neuronal death^60,61^. Early inhibition of iNOS and NOX immediately after injury prevents reactive gliosis and neuronal injury and protects the brain from oxidative damage^60,62^. In agreement with these studies, we found that nitro-oxidative stress markers were elevated in the neocortex of TBI mice and mitigated acutely and chronically with early naltrexone treatment. However, naltrexone has no effect on 3-NT levels at 3 months. The lack of effect on 3-NT levels after injury could be due to several reasons including inadequate dosage or treatment regimen, drug specificity, time window of treatment, brain’s compensatory mechanisms, complexity of injury pathology, individual variability, or methodological limitations.

Oxidative stress can regulate inflammatory genes by activating MAPKs. MAPK signaling is required for the expression of iNOS and of IL-6, IL-1β, and TNFα, all inflammatory cytokines with pivotal cellular roles^63,64^. The interaction between opioid receptors and the immune system is bidirectional: receptors can be immunosuppressive or immunostimulatory. In an in vitro culture model enriched for primary microglia, the stimulation of MOR increased the production of IL-1β, TNFα, IL-6, and nitric oxide, causing a reactive phenotype in these cells^13^. Reports have shown that chronic activation of MOR can activate microglia and astrocytes, releasing inflammatory cytokines^65^. Upregulation of these cytokines in glial and immune cells has also been linked to brain neurodegeneration in various chemo-convulsant models of temporal lobe epilepsy^66–68^. Likewise, in our experimentally injured mice, IL-1β, TNFα, and other cytokines were upregulated in the neocortex at early and late time points post-injury and, concurrently, their brains developed gliosis and neurodegeneration. At the same time, MOR protein and S375 phosphorylation levels also increased. Strikingly, inhibiting MOR with naltrexone reduced several but not all inflammatory cytokines/chemokines in the brain and serum and prevented neuroinflammation, suggesting that blocking MOR with naltrexone may play a role in reducing immune and neuroinflammation. However, some of the inflammatory markers such as cytokines remain unaffected at acute and chronic time-points. The response of cytokines after TBI is complex and dynamic, influenced by various factors such as injury severity, timing, and location, as well as individual differences in immune responses^69–71^. When studying TBI in animal models, researchers often aim to recapitulate the pathophysiological processes observed in human patients. However, discrepancies in the cytokine response between animal models and human patients may arise. While it might be expected that cytokine levels would increase after TBI due to inflammatory response, several factors could contribute to the lack of increase or even a decrease in certain cytokines. These factors include temporal dynamics of cytokines, compartmentalization of the immune response, regulatory mechanisms that modulate inflammatory response, heterogeneity of injury, individual variability, species differences, methodological considerations, model specificity, immune system complexity, resolution of inflammation etc^72–74^. While animal models serve as important tools for studying TBI and testing potential therapies, they may not fully replicate the cytokine response observed in human patients. Moreover, further research and better animal models are needed to elucidate the specific cytokine profiles associated with different types and severities of TBI and to identify potential therapeutic targets for modulating the immune response and promoting recovery.

TBI changes the anterior white matter structure broadly, which can be visualized through fractional anisotropy (FA)—a measure of anisotropic water diffusivity in the brain that provides a read-out of fiber organization, orientation, and the degree of myelination^75^. In various closed-head injury models, numerous neuroimaging studies reported widespread damage to the white-matter structures and discontinuity in fiber reconstructions in different brain regions after injury^76–78^. Studies on epilepsy models have also reported a decrease in FA in major bundle fibers, in the hemisphere ipsilateral to the seizure origin and in the corpus callosum, and in white matter adjacent to the neocortex and hippocampus^75,79,80^. Since trajectories of FA changes are likely predictive of outcomes after TBI, we used FA to evaluate changes in the whole brain after TBI and with naltrexone therapy. Consistent with the previous studies, we found reductions in FA after TBI, especially in the white matter adjacent to the neocortex, corpus callosum, and internal fiber tracts, both during the acute and chronic phases^81^. Since naltrexone was able to reduce changes in FA, which is a measure of axonal damage, this provides some evidence that naltrexone prevented further injury to the white matter. Some regions where naltrexone prevented axonal damage are the neocortex, peri-hippocampal fiber tracts, corpus callosum and thalamus. Persistent brain inflammation following a TBI causes tissue damage and white-matter degeneration that is commonly assessed using DTI^25,82^. Reports have shown that substantial number of people who have developed epilepsy after moderate to severe traumatic brain injury have non-lesioned MRI, meaning that the injury was a diffuse injury, and the axonal fibers are sensitive to the shearing and stretching forces that occur after TBI^26–28,83^. Perhaps by attenuating inflammation, naltrexone protects white matter from deterioration. Myelin is essential for the proper functioning of fiber tracts, as it facilitates the rapid transmission of nerve impulses. Certain anti-seizure drugs have been shown to promote myelin formation and repair in animal models of epilepsy^84^. By supporting myelin maintenance and repair processes, these drugs and probably naltrexone in this study, may help protect fiber tracts from degeneration. In addition, dysregulation of glutamate signaling has also been implicated in excitotoxicity and white matter damage in during seizures^85,86^. By modulating glutamate and GABA transmission, naltrexone may help maintain the balance of neurotransmitters activity within fiber tracts, thereby protecting them from excessive excitatory stimulation. Despite all such possibilities, the mechanisms by which naltrexone protects myelination and maintains white-matter fiber integrity in the brain are still unknown. Yet, we should also consider other factors, such as changes in diffusivity, cerebral blood flow autoregulation, blood–brain–barrier disruption, and genetic modulators. Additional studies are required to confirm the correlations of these factors with white-matter damage after the injury and to explore mechanisms underlying naltrexone’s neuroprotective effects.

Although the persistent occurrence of interictal spiking does not always lead to epilepsy, its occurrence over a long period of time can serve as a crucial diagnostic biomarker for epilepsy^87,88^. In our study, we observed interictal events over the twelve-week observation period after TBI. The twelve-week time-point was chosen based on some traumatic brain injury models of PTE and also based on the transmitter battery life^25,49,89,90^. Notably, these discharges were reduced by naltrexone, and seizures did not develop for up to 3 months in the naltrexone-treated group. While Naltrexone’s target, MOR, plays a known but incompletely understood protective role in epilepsy, this receptor subtype has also been implicated in the pro-convulsive actions of morphine^91,92^. Our observations provide evidence that MOR may function in the pro-convulsive capacity after TBI and that naltrexone can block this effect.

The increased spiking observed in the TBI group in our model could be linked, in principle, to either enhanced neuronal excitability or decreased inhibition. As MORs are widely expressed on GABAergic interneurons, they can reduce neuronal GABAergic activity, causing disinhibition^93–95^. This may facilitate excitatory signaling, thereby enhancing seizure susceptibility. Another plausible explanation is that MOR increases excitability in pyramidal cells by closing K+ channels and increasing NMDA receptor- and L-type Ca^2+^ channel-mediated Ca^2+^ entry^96,97^. In our prior publication studying naltrexone’s anti-convulsant effect in mouse brain slices and in a zebrafish model, seizure-like events were induced by blocking GABAergic activity with pentylenetetrazol^21^. Thus, our prior results suggest that naltrexone may act by decreasing excitability rather than altering inhibition.

Collectively, our previous and our current study indicate that naltrexone has anti-convulsive and anti-inflammatory properties. Despite this, our study does have some limitations that need to be addressed in future studies. First, better TBI models are required, including weight drop which is known to cause high variability of injury severity. Although, our method was modified to control the magnitude and location of the impact that leads to variability, we still need more reliable WD and other TBI models that can closely replicate the complexity of TBI in humans. Second, the duration over which post-traumatic seizures were evaluated in our study, as longer post-TBI periods are required to better assess the post-traumatic epilepsy conditions. Third, while animal models provide valuable insights into TBI pathology and potential treatments, findings from the weight drop model may not always directly translate to human TBI due to interspecies differences in brain anatomy, physiology, and injury response. Fourth, the weight drop model often focuses on acute effects of TBI and may not adequately address the long-term consequences and chronic pathology observed in human TBI survivors. Fifth, our study does not answer whether naltrexone’s neuroprotective effect is due to decreased inflammation or due to directly altered neuronal excitability, or by a combination of both.

In conclusion, our findings illustrate the neuroprotective effects of naltrexone in a mouse model of post-traumatic seizures when the drug was administered starting one day after the brain injury. Mechanistically, naltrexone has strong anti-inflammatory properties and reduces neuroinflammation by targeting reactive gliosis, oxidative stress (ROS/RNS), inflammatory cytokine production, neurodegeneration, and epileptiform discharges and, consequently, modifying the development of post-traumatic seizures.

## Methods

### Experimental groups, induction of traumatic brain injury, and drug treatment

**Figure 8:**
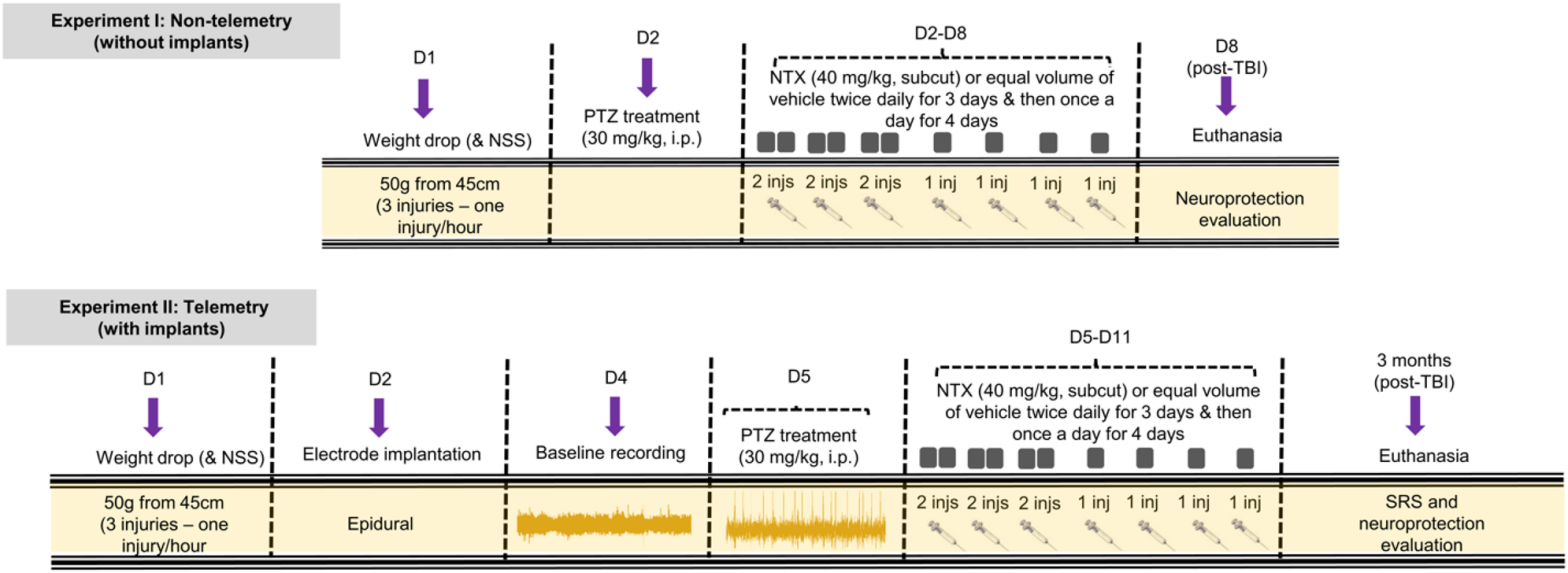
TBI model and naltrexone treatment regimen. Experimental design illustrating non-telemetry (experiment I, without implants) and telemetry (experiment II, with implants) groups, and detailing their respective treatment regimens and endpoints. For non-telemetry animals, traumatic brain injury was induced on day 1 (D1) and the PTZ-test was performed on day 2, whereas for telemetry animals, the PTZ-test was performed on day 5 post-TBI, i.e., after the electrode implantation (day 2). Naltrexone treatment was initiated 2 h after administration of the sub-convulsive dose of PTZ. The monitoring of the spontaneous seizures (experiment II) started three weeks after TBI. Animals were euthanized at 8 d (for experiment I) and 3 month (for experiment II) post-TBI.

The source of the Naltrexone was obtained from Tocris Bioscience. Mice (n = 95) were randomly divided into four groups. Group I, sham (S), received only naltrexone (no TBI); Groups II (TBI) and III sustained a TBI, after which Group III received naltrexone (T + N); and Group IV received naltrexone, without TBI (NTX group). To induce TBI, a Marmarou WD model was employed on a four-week-old male C57BL/6J mice, with slight modifications to minimize variability^98,99^. Animals were habituated for 2 h prior to injury and mildly anesthetized using gaseous isoflurane. Mice were anesthetized for five minutes and the depth of anesthesia was assessed by a toe pinch test. An eye ointment was applied, and 1 mg/kg meloxicam was administered subcutaneously prior to WD. The mouse head was positioned under the weight drop tube and a flat stainless-steel disc was placed in the center of the head. A free fall weight of 50 g was dropped on the sagittal line between the coronal and lambdoidal sutures from the height of 45 cm so that the weight would impact the midline of the skull between the ears. To adjust this area, the area between the line passing behind the eyes and the ear was marked with a light source placed on top of the stainless-steel tube. The weight and height of the impact were standardized based on our pilot studies, taking mortality and injury severity into account (Supporting Figs. 1 and 2). Post-impact, animals were immediately placed on a heating pad, and the time taken by each mouse to regain consciousness was recorded. One hour after injury, we validated the modified Marmarou WD by using two parameters: loss of consciousness (LOC) and Neurological Severity Score (NSS)^100–102^. Our results indicated that compared to the controls, there was a significant difference in both LOC and NSS following a WD utilizing the modified Marmarou (Supporting Fig. 2). At the end of the assessment, softgel, Nutrical and 0.5 mL saline was given to all the animals. For non-telemetry animals (without electrodes), a second treatment, referred to as the PTZ test, was performed on day 2 post-TBI, and for telemetry animals (with electrodes), the PTZ test was performed on day 5 post-TBI (Fig. 8). The PTZ test consisted of a sub convulsive dose of PTZ (30 mg/kg, i.p.), given as a second “hit” after the initial weight drop.

Naltrexone treatment (40 mg/kg, subcutaneous) was initiated two hours after PTZ injection, and was continued every 12 h for three days and then once daily for four days (Fig. 8)^21^. The choices of dosage, route, and treatment regimen were decided based on our pilot data, the toxicological and drug safety profile studies and the drug’s pharmacological properties^103–109^. EEG was recorded continuously for 8 days after TBI, and then intermittently (8 h/day) for up to 3 months. Animals were euthanized at 8 d and 3 months post-TBI. PTZ and naltrexone were freshly prepared in saline at a 5 mg/mL concentration. All experiments on animals were performed under the Institutional Animal Care and Use Committee guidelines, University of Iowa, USA.

### Electrode implantation, radiotelemetry setup and video-EEG recording

The detailed procedure for electrode implantation, radiotelemetry setup and recording are described in our previous and recent publications^42,43,48,110^. Mice were placed on a heating pad and anesthetized using gaseous isoflurane. The head was shaved, artificial tear ointment was applied (Aventix, CAT: 13585) and meloxicam (1 mg/kg, subcutaneously) was administered prior to surgery. A midline incision was made from the mid-dorsal region of the head toward the neck to expose the skull. A subcutaneous pocket was created in the flanking region for the HD-X02 PhysioTel™ radio-transmitter (Data Science International, CAT: 270-0172-001). Bilateral burr holes were drilled into each hemisphere of the brain and two electrodes were placed epidurally in a V-shaped pattern. The electrodes were secured in place using dental cement. Surgical clips and sutures were used to close the incision. After surgery, antibiotic Baytril (5 mg/kg, subcutaneous) was given to prevent bacterial infection. Topical triple antibiotic ointment was applied on the incision site, and 1 ml saline was given to replenish lost electrolytes. Mice were then placed in an empty cage on a heated pad until they regained consciousness and became ambulatory. One day after electrode implantation, mice were moved into the telemetry room and 24 h of baseline EEG was recorded using a PhysioTel™ radiotelemetry device. Animals were individually caged and placed on PhysiolTel™ receiver model RPC-1 receiving pads. Pads were spaced out on tables with aluminum shields placed between pads to eliminate signal crossing between pads. These receiving pads were connected to a Matrix 2.0 for PhysiolTel™ for data exchange with the PC through Ponemah, version 6.51. Four AXIS M1145-L Network Cam Kits and Media Recorder 4 software, version 4.0, were used to capture video to help create eight video-synchronized EEG traces. One camera kit was able to record two receiver pads at a time. The Ponemah software combines all data from the Media Recorder software and the Matrix 2.0 to create video-synchronized, two channel, EEG traces with the sampling frequency of 1000 Hz with a filter cutoff of 100 Hz and full scale at 10 mV. Videos were captured at 20 frames/second with a resolution of 1280 × 720. Files were saved directly onto a 4 TB Seagate hard drive. Following the acclimation period and baseline recording, the PTZ test was performed by administering a sub-convulsive dose of 30 mg/kg intraperitoneally. Following one hour of behavioral observation (or 2 h post-PTZ dose), naltrexone (40 mg/kg, subcutaneous) or vehicle treatment was initiated and was given twice a day for three days and then once a day for four days. For the initial phase of EEG recording (the first seven days following TBI by weight drop), video-synchronized EEG was recorded for 24 h/day, and then, in the subsequent phase, EEG was recorded intermittently for up to three months. For this period, EEG was recorded for 8–10 h a day for five days a week, followed by video recordings overnight and over the weekends. All the seizures were verified against the power spectrum (high-frequency gamma bands), electromyogram, and the videos synchronized with EEG.

### Quantification of electrographic spikes and seizures

We developed a novel MATLAB-based algorithm that analyzes spiking activity and interictal events to quantify mouse EEG data. Mouse EEG data was imported as an EDF file into MATLAB. Baseline EEG activity was determined autonomously for each individual data file. The lower threshold for spike detection was specified as twice the baseline activity^111^. The upper threshold for spike detection was set at 1500 μV, as spikes larger than 1500 μV often arise from electrical interference^47,48^. Next, EEG spikes were identified by the algorithm and were passed through a set of filters to remove any spike that failed to meet the spiking criteria (minimum peak height of 200 µV; maximum peak width of 200 ms). The remaining algorithm code quantified interictal events by analyzing clusters of spiking activity. These events were again passed through the filters, removing any event that failed to meet the interictal event criteria^48,112^. Each spike in an event has amplitude 3X times the baseline, with > 3 spikes in an event and an inter-spike interval of 10 s^44,112^. Electrographic seizures were also identified through a similar pipeline, i.e., lower detection threshold was twice the baseline, upper detection ≤ 1500 μV and an inter-spike interval of ≥ 100 ms. The electrographic seizures were manually and visually reviewed using Neuroscore software. The EEG data was then run through our automated algorithm capable of detecting electrographic seizures as a second test to validate our results. The detailed procedure for our algorithm-based automatic quantification of electrographic spikes and seizures has been published recently^110^.

### Immunohistochemistry, microscopic imaging, and cell quantification

Animals were transcardially perfused initially with 1XPBS for five minutes, followed by 4% paraformaldehyde (PFA) at 8 days and 3 months post-TBI. Blood was collected, stored at room temperature (RT) for 10 min, spun at 2000 g at 4 °C, and serum was collected. After fixation, brains were dissected out and post-fixed in 4% PFA overnight at 4 °C. The following day, fixed brains were cryopreserved in 30% sucrose for 3–4 days at 4 °C or until they settled to the bottom of the vial. All tissues were then embedded in gelatin type B, wrapped in saran wrap and stored overnight at 4 °C. The next day, brain blocks were prepared by snap-freezing the gelatin-embedded tissue blocks in liquid nitrogen, using 2-methylbutane, and stored at − 80 °C. Brain blocks were serially sectioned into 12 µm thickness sections using a CryoStar NX70 cryostat with 3–4 sections/slide mounted sequentially at approximately 375 µm apart, representing rostral to caudal parts of the brain. The section sampling and collection method has been described in detail by Puttachary et al. (2016). For immunohistochemistry (IHC), sections were washed with 1XPBS for 45 min and then blocked with 10% donkey serum containing 0.2% Triton X-100. After blocking for an hour at room temperature (RT), brain sections were stained with primary antibodies of interest and were incubated overnight at 4 °C. The following day, after washing with 1XPBS, sections were immunostained with the appropriate secondary antibody (FITC- or CY3-conjugated) for an hour at RT, washed with 1XPBS again and then were mounted with vectashield 4’,6-diamidino-2-pheny-lindole (DAPI) for nuclear staining.^42–44^ Descriptions and dilutions of the primary and secondary antibodies are listed in Supporting Table 1. To determine the extent of neurodegeneration, we performed Fluorojade B staining (FJB), co-immunostained with NeuN. For FJB staining, sections were incubated in 0.006% potassium permanganate solution for 10 min. After washing three times with distilled water (30 s for each wash), slides were submerged in 0.001% FJB + 0.1% acetic acid solution for 10 min in the dark. After washing, slides were air-dried in the dark at room temperature, cleared with xylene and mounted with Surgipath Acrytol (Surgipath, Leica Biosystems, IL)^42–44^. To eliminate variability, brain sections from all the groups were prepared and stained simultaneously.

Images were taken using a Hamamatsu C13440 Digital Camera, ORCA-Flash 4.0 and Pika Allies vision, on an Olympus virtual slide scanner with a 4X DAPI overview scan. FITC and TRITC exposures were 101.45 ms and DAPI exposure was 70 ms, and the images of all the regions of interest (ROI) were taken at 20X. Both left and right cortex were the ROI used for the study and quantification. All the images were counted bilaterally and manually from 3–4 sections/animal at bregma level -1.45, -1.95, and -2.35 at 20X magnification using ImageJ. For magnification, multiple focus points were placed within the ROIs to attain clear pictures by adjusting the clarity points manually. After the slides were scanned, images were converted to TIFF files for quantification using ImageJ. All the images were counted bilaterally and manually from 3–4 sections/animal at bregma levels − 1.45, − 1.95, and − 2.35 at 20× magnification using ImageJ from a known area. IHC data was expressed as positive cells/mm^2^ and the outcome determined by averaging the total number of cells among the sections.

### Western blotting

The cortical tissues were dissected from the mice at 8 days and 3 months post-TBI and snap-frozen in liquid nitrogen. Tissues were homogenized and lysed in NET100 buffer containing 5 M NaCl_2_, 0.5 M EDTA and 1 M Tris pH 8 with 1% protease and phosphatase inhibitor (ThermoFischer Scientific, USA). The supernatant was collected and stored at − 80 °C for later analysis. The protein concentrations from tissue lysates were determined using the Bradford assay kit (Biorad, USA; Cat# 5000006). Equal amounts of protein (30 μg) were loaded in the wells of 7.5–10% precast denaturing polyacrylamide gels along with a molecular weight marker. The gels were run at RT at 80 V for 1 h, and then at 120 V until the bromophenol dye reached at least 0.5 cm from the bottom of the plate. Protein transfer to the nitrocellulose membrane was carried out by rapid transfer: the transfer sandwich containing the gel and membrane was placed into a Trans-Blot Turbo Transfer System (Biorad, USA), which was then run at 25 V for 9 min. Next, the membrane was washed with 1XTris-Buffered Saline (TBS) for 10 min and transferred to 5% nonfat dry milk in Tris-Buffered Saline with Tween 20 (1XTBST) (Sigma; cat# T-9039) for blocking for 1 h at RT. After the blocking step, the blots were incubated with primary antibodies of interest overnight at 4 °C. Descriptions and dilutions of the primary antibodies are listed in Supporting Table 1. The following day, the membranes were washed with TBST and incubated with Peroxidase AffiniPure anti-rabbit and mouse IgG antibodies (1:10,000, Jackson ImmunoResearch, USA) for 1 h at RT followed by further washes with TBST as described earlier. 5% milk and/or 5% BSA in TBST was used as a diluent for both primary and secondary antibodies. Protein bands were detected using SuperSignal™ West Pico Plus chemiluminescent substrate and identified using a MyECL imager. All bands were normalized against β-actin and quantified using densitometric analysis on Image J.

### Quantitative real time-polymerase chain reaction

Gene expression of inflammatory cytokines was determined by quantitative real-time PCR. RNA was extracted using the trizol-chloroform method per manufacturer’s instructions (ThermoFisher Scientific). 1 µg of RNA was used for cDNA synthesis using SuperScript® III First-Strand Synthesis kit, yielding high-quality single-stranded cDNA. cDNA synthesis reactions were performed as follows: primer annealing at 70 °C, 10 min; hold at 15 °C for 5 min; then reverse transcription at 42 °C, 50 min; 70 °C for 15 min and hold at 15 °C. The following day, plate reactions were prepared using amplified cDNA diluted in ultrapure water (1:15) with a 2× universal master mix. Plates were run for quantitative real-time PCR (qRT-PCR) using pre-validated qPCR primers for the genes of interest (Supporting Table 1) on a QuantStudio™ 7 Pro using FAM-MGB_VIC-Tamra fluorescent labels, as follows: 10 min, 95 °C; then 95 °C for 15 s, 60 °C for 1 min (40 cycles). GAPDH was used to normalize all the genes. The fold change in mRNA expression was determined using cycle threshold (Ct) values, and results were expressed as fold difference from controls.

### Multiplex cytokine assay

A multiplex assay using a Quantibody Custom Mouse Cytokine Array (QCMCA) was performed by RayBiotech, on the serum samples from 8 day and 3-month post-TBI mice (CAT: QAA-CUST, RayBiotech Inc, GA, USA). The core QCMCA technology uses glass slides onto which protein targets are attached in an array format, allowing for multiple detection of 32 (or more) analytes in the same sample at a given time. Briefly, cytokine standard dilutions were prepared from a lyophilized cytokine standard mix after reconstituting with 500 μl sample diluent (standard 1). 200 µL of sample diluent was added to six different microcentrifuge tubes (standards 2–7). Serial dilutions were performed by adding 100 μl of standard 1 to standard 2, and then from standard 2 to tube 3, and so on. For blocking, 100 μl of sample diluent was added to each well and incubated for 30 min. After the buffer was decanted, 100 μl of standard cytokines or samples were added and incubated at RT for 1–2 h. Post-incubation, samples were washed five times for five minutes each with 150 μl of 1X wash buffer. Detection antibody was reconstituted with 1.4 mL of sample diluent, and 80 μl of detection antibody cocktail was added to each well and incubated at RT for 1–2 h. After incubation, samples were decanted, and each well was washed with 150 μL of 1X Wash Buffer I and twice with 150 μL of 1X Wash Buffer II. After washing, 80 μL of dye-conjugated streptavidin was added to each well, covered with aluminum foil, and incubated for an hour at RT. Wells were washed five times using wash/drying/wash cycles with 1X Wash Buffer I and II. Signals were visualized with a laser scanner equipped with Cy3 wavelength channel such as Axon GenePix or Innopsys Innoscan. Data were extracted using GAL files and converted to Excel files.

### Acquisition and analysis of diffusion-weighed MRI

#### Image acquisition

Mice from both groups, TBI and T + N, were scanned before TBI, and at 8 days and 3 months post-TBI. Imaging was performed on a GE/Agilent Discovery 901 7-Telsa pre-clinical scanner. Mice were anesthetized with isoflurane (3% induction, 1.5% maintenance) in oxygen, and depth of sedation was monitored via the respiratory rate with an MR-compatible monitoring system (SA Instruments, Inc., New York). MRI imaging acquisitions included a high-resolution 3D anatomical scan to derive structural information and a 32-direction diffusion tensor imaging (DTI) scan to investigate microstructural integrity. The 3D anatomical scan was acquired with three-dimensional fast imaging employing a steady-state acquisition pulse sequence. A 224 × 192 × 112 matrix was acquired over a 25 mm × 25 mm × 20 mm field of view resulting in a native voxel resolution of 0.11 mm × 0.13 mm × 0.18 mm at a TR/TE = 5.8 ms/2.8 ms, flip angle 30°, and 3 signal averages. The diffusion-weighed scan utilized an 8-shot 2D segmented echo-planar imaging (EPI) to a 128 × 128 matrix over a 25 mm × 25 mm field of view with 20 slices at 0.8 mm thickness. The scan acquired 15 diffusion directions with b = 1000 s/mm^2^ along with 2 T2 (b = 0) images with TR/TE = 4000 ms/18.4 ms.

#### Image preprocessing and TBSS analysis

After acquisition, diffusion-weighed images were converted from DICOM to NIFTI format using DCM2NIIX and examined for quality. Bias field correction was applied using N4BiasFieldCorrection from Advanced Normalization Tools (ANTs), and images were resampled into a voxel resolution of 0.2 mm^3^ for use in the processing pipeline^113^. We performed voxel-wise statistical analysis of collected fractional anisotropy (FA) data using a version of Tract-Based Spatial Statistics (TBSS), from FSL developed in house and optimized for mouse imaging^113,114^. Using the diffusion toolbox (FDT), a tensor model was fit to the diffusion data to generate FA images, which were brain-extracted using hand-drawn masks. FA data for all animals were then aligned into Waxholm space using the nonlinear registration tool FNIRT from FSL^115^. Next, mean FA images were created and thresholded at a value of 0.2 to delineate major fiber tracts and create a mean FA skeleton representative of the centers of all fiber tracts in the data. Each subject’s aligned FA data was projected onto the mean FA skeleton, and the resulting data was used to perform voxel-wise cross-subject statistics using the randomize tool from FSL, with 252 permutations and threshold-free cluster enhancement (TFCE) at *p* < 0.05 to check for significance in the contrasts.

#### Methodological rigor

The experimenters were blind to the experimental groups until after the data analyses were completed. We followed pre-determined criteria to exclude animals from the data analyses. The criteria set were: (1) if animals did not respond to the predetermined injury (increased loss of consciousness [LOC] and neurological severity score [NSS] compared to sham); (2) if animals died during the course of the experiment; and (3) if animals did not regain bodyweight within 8–10 days and if EEG electrodes were displaced during the recording period. In total, only four mice were excluded from the study. Three of these mice had post-surgical complications after electrode implantation and did not gain body weight after surgery. One mouse died due to seizure during the EEG recording period. Exclusion criteria for mice into the study was determined based on three parameters following WD: LOC, NSS and PTZ susceptibility. From preliminary studies we determined WD from 45 cm induced an LOC averaging 120–140 s with an NSS score averaging 5 without compromising mortality (Supporting Fig. 2). This ensured only mice with a severe impact continued onto the PTZ test and if any mice were susceptible at a sub convulsive dose of 30 mg/kg they were included in the study. We also took measures to minimize variables by: (1) randomizing the animals based on a predetermined weight (≥ 12 g mice) and age range (4 weeks) before the start of the experiment; (2) quantifying injury severity during the weight drop by both direct observation and offline video analysis and using at least two independent observers; (4) acquiring ~ 24 h of baseline EEG data, covering day-night cycles, to normalize post-TBI EEG from the same animal; (4) where appropriate, implementing the first two of the three principles of reduction, refinement, and replacement (3Rs) by adopting a refined, predetermined method of TBI induction, which reduced the mortality rate and minimized variability in TBI severity between animals and groups; and, (5) determining the optimum concentration of the primary antibodies by serial dilution and validating their specificity using neutralizing antibodies appropriate to the primary antibodies. All methods performed on mice were in accordance with the ARRIVE guidelines.

### Statistical analyses

Normality of the data was tested using the Shapiro–Wilk and Kolmogorov–Smirnov tests. Unpaired *t*-tests were used for parametric comparisons, while the Mann–Whitney U was used for unpaired non-parametric comparison. One-way repeated analysis of variance (ANOVA) was used for multiple comparisons of parametric data with Tukey’s post-hoc analysis. For spike analysis, two-way ANOVA (or mixed-effects model) with Geisser-Greenhouse correction was used for multiple comparisons with the Sidak post-hoc test. PTS incidence analysis was performed utilizing Kaplan–Meier analysis with log rank test. Statistical significance was set to *p* < 0.05. Prism 9 was used for data analysis.

### Study approval

Animal procedures were performed in accordance with NIH guidelines and approval from IACUC of the University of Iowa.

### Supplementary Information

Supplementary Information.

## Data Availability

Data will be available upon request. Supportive analytic code is available here https://github.com/Jackson-Kyle-CCOM/Automated-EEG-Algorithm.
