# Supplementary material for: Neuroprotective effects of naltrexone in a mouse model of post-traumatic seizures
Source: Sci Rep. 2024 Jun 12;14:13507. doi: 10.1038/s41598-024-63942-8 (PMC11169394; doi:10.1038/s41598-024-63942-8)
Supplement: Supplementary file 1 — Supplementary Information. [file 41598_2024_63942_MOESM1_ESM.pdf]

Supporting Data

Neuroprotective effects of naltrexone in a mouse model of post-traumatic seizures

Saul Rodriguez, Shaunik Sharma, Grant Tiarks, Zeru Peterson, Kyle Jackson, Daniel Thedens, Angela Wong, David Keffala-Gerhard, Vinit B. Mahajan, Polly J. Ferguson, Elizabeth Newell, Joseph Glykys, Thomas Nickl-Jockschat, Alexander G. Bassuk

Table of contents

Supporting Tables

Supporting Table 1 Reagents Table
Supporting Table 2 Serum multiplex cytokine assay table
Supporting Table 3 Quantification table for NeuN positive cells
Supporting Table 4 Fractional anisotropy analysis
Supporting Table 5 Number of spontaneous seizures with seizure stage

Supporting Figures

Supporting Figure 1 Standardization of surgical and drug administration protocol after TBI
Supporting Figure 2 Neurobehavioral status of mice after TBI

Raw Western Blots

Western Blot 1 S375
Western Blot 2 MOR
Western Blot 3 Phospho-p38
Western Blot 4 3-NT
Western Blot 5 iNOS

Supporting Table 1. Reagents

Antibody	Species/Clone	Used For	Dilution	Catalog #	Vendor
Anti-MOR	Rabbit polyclonal	IHC	1:1500	AB1580-I	Millipore Sigma
Anti-IBA1	Goat polyclonal	IHC	1:400	NB100-1028	Novus Biologicals
Anti-NeuN	Rabbit polyclonal	IHC	1:400	ab104225	Abcam
Anti-MOR	Rabbit polyclonal	WB	1:1000	AB1580-I	Millipore Sigma
Anti-Phosphor-p38 MAPK	Rabbit monoclonal	WB	1:1000	4511S	Cell Signaling
Anti-3-NT	Mouse monoclonal	WB	1:1000	ab61392	Abcam
Anti-iNOS	Rabbit polyclonal	WB	1:800	NB300-605	Novus Biologicals
Anti-β-actin	Mouse monoclonal	WB	1:5000	A2228	Sigma-Aldrich
FITC-conjugated	Anti-goat	IHC	1:50	705-095-147	Jackson Immunoresearch
FITC-conjugated	Anti-Mouse	IHC	1:80	715-095-150	Jackson Immunoresearch
FITC-conjugated	Anti-rabbit	IHC	1:100	711-095-152	Jackson Immunoresearch
CY3-conjugated	Anti-rabbit	IHC	1:200	711-165-152	Jackson Immunoresearch
CY3-conjugated	Anti-goat	IHC	1:200	705-165-147	Jackson Immunoresearch
Peroxidase AffiniPure Goat Anti-rabbit	Anti-rabbit	WB	1:10000	111-035-144	Jackson ImmunoResearch
Peroxidase AffiniPure Goat Anti-mouse	Anti-mouse	WB	1:10000	115-035-003	Jackson ImmunoResearch

Primer	Used For	Catalog #	Vendor
IL-1β	qPCR	Mm00434228_m1	ThermoFisher Scientific
TNF-α	qPCR	Mm443258_m1	ThermoFisher Scientific
IFNγ	qPCR	Mm01168134_m1	ThermoFisher Scientific
C3	qPCR	Mm01232779_m1	ThermoFisher Scientific
IL-12A	qPCR	Mm00414165_m1	ThermoFisher Scientific
IL-12B	qPCR	Mm00434174_m1	ThermoFisher Scientific

Primary and secondary antibodies used for immunohistochemistry and Western blotting. Gene primers used for Quantitative real-time PCR.

Supporting Table 2. Multiplex cytokine assay on serum at 8 days and 3 months post-TBI

Cytokine	Treatment Groups	p-value	Mean difference	95% CI of difference	DF	Sample size
**BAFF (8D)**	Sham vs TBI	0.2590	167.1	-75.47 to 409.6	28	8, 8
	TBI vs TBI+NTX	0.9726	-38.32	-280.8 to 204.2	28	8, 8
**BAFF (3M)**	Sham vs TBI	0.0340*	385.9	22.88 to 748.9	28	8, 8
	TBI vs TBI+NTX	0.2743	-245.3	-608.3 to 117.7	28	8, 8
**BTC (8D)**	Sham vs TBI	0.4933	1.117	-1.128 to 3.361	14	6, 6
	TBI vs TBI+NTX	>0.9999	0.01667	-2.732 to 2.765	14	6, 3
**BTC (3M)**	Sham vs TBI	0.5915	1.074	-1.354 to 3.502	15	5, 7
	TBI vs TBI+NTX	0.5494	-1.214	-3.813 to 1.385	15	7, 4
**Eotaxin (8D)**	Sham vs TBI	0.0030**	-26.96	-45.86 to -8.063	28	8, 8
	TBI vs TBI+NTX	0.5363	9.388	-9.512 to 28.29	28	8, 8
**Eotaxin (3M)**	Sham vs TBI	0.0033**	-19.03	-32.52 to -5.531	28	8, 8
	TBI vs TBI+NTX	0.0024**	-19.64	-33.13 to -6.144	28	8, 8
**IL-2Ra (8D)**	Sham vs TBI	0.0231*	-43.95	-83.03 to -4.870	27	8, 8
	TBI vs TBI+NTX	0.7089	-15.85	-56.30 to 24.60	27	8, 7
**IL-2Ra (3M)**	Sham vs TBI	0.0024**	-57.44	-96.75 to -18.13	27	8, 8
	TBI vs TBI+NTX	0.9508	7.625	-31.69 to 46.94	27	8, 8
**IL-28 (8D)**	Sham vs TBI	0.1512	4.817	-1.271 to 10.90	18	8, 6
	TBI vs TBI+NTX	>0.9999	-0.01667	-7.987 to 7.954	18	6, 3
**IL-28 (3M)**	Sham vs TBI	0.9853	-0.5625	-5.109 to 3.984	20	8, 8
	TBI vs TBI+NTX	0.6272	-2.125	-7.036 to 2.786	20	8, 6
**IL-33 (8D)**	Sham vs TBI	0.9134	4.533	-14.75 to 23.82	22	6, 7
	TBI vs TBI+NTX	0.9977	1.340	-18.96 to 21.64	22	7, 5
**IL-33 (3M)**	Sham vs TBI	0.5378	-4.452	-13.65 to 4.748	19	8, 7
	TBI vs TBI+NTX	0.9935	1.039	-10.10 to 12.18	19	7, 4
**CXCL10 (8D)**	Sham vs TBI	0.6307	-1084	-3809 to 1641	10	4, 6
	TBI vs TBI+NTX	0.6993	1236	-2211 to 4682	10	6, 2

**CXCL10 (3M)**	Sham vs TBI	0.6267	-948.7	-3258 to 1360	12	3, 5
	TBI vs TBI+NTX	0.9818	-249.6	-2249 to 1750	12	5, 5
**CXCL1 (8D)**	Sham vs TBI	0.7851	1.325	-2.534 to 5.184	28	8, 8
	TBI vs TBI+NTX	0.8557	1.125	-2.734 to 4.984	28	8, 8
**CXCL1(3M)**	Sham vs TBI	0.0005***	4.050	1.645 to 6.455	28	8, 8
	TBI vs TBI+NTX	0.0977	-2.125	-4.530 to 0.2795	28	8, 8
**Leptin (8D)**	Sham vs TBI	0.0468*	-203.3	-404.4 to -2.183	27	8, 8
	TBI vs TBI+NTX	0.7627	-74.44	-282.6 to 133.7	27	8, 7
**Leptin (3M)**	Sham vs TBI	0.5447	-102.5	-310.9 to 106.0	28	8, 8
	TBI vs TBI+NTX	0.0333*	222.3	13.83 to 430.7	28	8, 8
**CXCL5 (8D)**	Sham vs TBI	>0.9999	-0.3750	-60.06 to 59.31	28	8, 8
	TBI vs TBI+NTX	0.5037	30.83	-28.86 to 90.51	28	8, 8
**CXCL5 (3M)**	Sham vs TBI	>0.9999	0.4250	-48.49 to 49.34	28	8, 8
	TBI vs TBI+NTX	0.0975	-43.25	-92.16 to 5.664	28	8, 8
**CCL7 (8D)**	Sham vs TBI	0.0882	9.425	-1.021 to 19.87	28	8, 8
	TBI vs TBI+NTX	0.4474	5.763	-4.684 to 16.21	28	8, 8
**CCL7 (3M)**	Sham vs TBI	<0.0001****	51.89	30.73 to 73.04	28	8, 8
	TBI vs TBI+NTX	0.5833	-9.913	-31.07 to 11.24	28	8, 8
**RANK L (8D)**	Sham vs TBI	0.9981	-5.525	-92.63 to 81.58	26	8, 8
	TBI vs TBI+NTX	0.7655	32.00	-58.16 to 122.2	26	8, 7
**RANK L (3M)**	Sham vs TBI	0.0072**	-64.27	-113.5 to -15.02	25	6, 8
	TBI vs TBI+NTX	0.0055**	63.52	16.33 to 110.7	25	8, 7
**GM-CSF (8D)**	Sham vs TBI	0.9262	-3.625	-19.70 to 12.45	28	8, 8
	TBI vs TBI+NTX	0.4711	-8.625	-24.70 to 7.447	28	8, 8
**GM-CSF (3M)**	Sham vs TBI	0.6175	-7.193	-23.35 to 8.962	25	7, 8
	TBI vs TBI+NTX	0.0732	15.09	-1.062 to 31.25	25	8, 7
**IFN-γ (8D)**	Sham vs TBI	0.0089**	-36.10	-64.56 to -7.643	28	8, 8
	TBI vs TBI+NTX	0.9670	4.800	-23.66 to 33.26	28	8, 8
**IFN-γ (3M)**	Sham vs TBI	0.1956	-44.71	-104.6 to 15.15	25	8, 7
	TBI vs TBI+NTX	0.4671	33.14	-28.68 to 94.96	25	7, 7
**IL-1β (8D)**	Sham vs TBI	0.9463	-9.825	-59.66 to 40.01	22	8, 8
	TBI vs TBI+NTX	0.4636	-28.77	-82.60 to 25.06	22	8, 6
**IL-1β (3M)**	Sham vs TBI	0.8511	21.00	-51.40 to 93.14	23	8, 8
	TBI vs TBI+NTX	0.9057	-18.99	-96.91 to 58.93	23	8, 6
**IL-2 (8D)**	Sham vs TBI	0.8496	-1.838	-8.068 to 4.393	26	8, 8
	TBI vs TBI+NTX	0.9964	-0.5071	-6.956 to 5.942	26	8, 7
**IL-2 (3M)**	Sham vs TBI	0.8777	1.735	-4.686 to 8.156	24	5, 8
	TBI vs TBI+NTX	0.2478	3.913	-1.719 to 9.544	24	8, 8
**IL-3 (8D)**	Sham vs TBI	0.9998	-0.1875	-6.083 to 5.708	28	8, 8
	TBI vs TBI+NTX	>0.9999	0.08750	-5.808 to 5.983	28	8, 8
**IL-3 (3M)**	Sham vs TBI	0.3039	-2.875	-7.292 to 1.542	27	8, 8
	TBI vs TBI+NTX	0.2463	3.196	-1.375 to 7.768	27	8, 7
**IL-5 (8D)**	Sham vs TBI	0.9263	6.425	-22.08 to 34.93	28	8, 8
	TBI vs TBI+NTX	0.9789	-4.113	-32.62 to 24.39	28	8, 8
**IL-5 (3M)**	Sham vs TBI	0.9404	-12.89	-75.02 to 49.25	26	8, 8
	TBI vs TBI+NTX	0.6949	-25.70	-90.02 to 38.62	26	8, 7
**IL-9 (8D)**	Sham vs TBI	0.9373	-5.375	-30.68 to 19.93	28	8, 8
	TBI vs TBI+NTX	0.9830	3.388	-21.92 to 28.70	28	8, 8
**IL-9 (3M)**	Sham vs TBI	0.3241	-18.10	-46.63 to 10.43	26	8, 8
	TBI vs TBI+NTX	0.9948	-2.620	-32.15 to 25.91	26	8, 7
**IL-10 (8D)**	Sham vs TBI	0.3441	32.95	-20.14 to 86.04	27	7, 8
	TBI vs TBI+NTX	0.5381	-25.38	-76.67 to 25.92	27	8, 8
**IL-10 (3M)**	Sham vs TBI	0.6895	-19.45	-67.98 to 29.08	24	8, 8
	TBI vs TBI+NTX	0.0162*	-59.45	-109.7 to -9.220	24	8, 7
**IL-13 (8D)**	Sham vs TBI	0.6290	11.25	-16.60 to 39.63	12	6, 2
	TBI vs TBI+NTX	0.9885	-3.070	-31.88 to 25.74	12	2, 5

**IL-13 (3M)**	Sham vs TBI	0.5779	-8.025	-26.51 to 10.46	11	4, 6
	TBI vs TBI+NTX	0.9775	2.683	-17.57 to 22.94	11	6, 3
**IL-17 (8D)**	Sham vs TBI	0.3626	-3.413	-9.022 to 2.197	28	8, 8
	TBI vs TBI+NTX	0.9448	1.138	-4.472 to 6.747	28	8, 8
**IL-17 (3M)**	Sham vs TBI	0.9791	-1.100	-8.797 to 6.597	26	8, 8
	TBI vs TBI+NTX	0.5706	-3.650	-11.35 to 4.047	26	8, 8
**CCL2 (8D)**	Sham vs TBI	0.2548	11.39	-5.056 to 27.83	28	8, 8
	TBI vs TBI+NTX	0.7678	-5.838	-22.28 to 10.61	28	8, 8
**CCL2 (3M)**	Sham vs TBI	0.9819	2.250	-14.27 to 18.77	27	8, 8
	TBI vs TBI+NTX	0.2753	-11.52	-28.62 to 5.573	27	8, 7
**M-CSF (8D)**	Sham vs TBI	0.0074**	3.263	0.7451 to 5.780	27	8, 8
	TBI vs TBI+NTX	0.8508	-0.7679	-3.374 to 1.838	27	8, 7
**M-CSF (3M)**	Sham vs TBI	0.4855	-1.138	-3.293 to 1.018	28	8, 8
	TBI vs TBI+NTX	0.7520	-0.7875	-2.943 to 1.368	28	8, 8
**VEGF (8D)**	Sham vs TBI	0.0397*	77.90	2.847 to 153.0	28	8, 8
	TBI vs TBI+NTX	0.2347	-53.34	-128.4 to 21.72	28	8, 8
**VEGF (3M)**	Sham vs TBI	0.6103	47.38	-57.33 to 152.1	28	8, 8
	TBI vs TBI+NTX	0.2815	-70.14	-174.8 to 34.57	28	8, 8
**IL-1α (8D)**	Sham vs TBI	0.0018**	-0.8262	-1.370 to -0.2825	23	6, 7
	TBI vs TBI+NTX	0.0061**	0.7262	0.1825 to 1.270	23	7, 6
**IL-1α (3M)**	Sham vs TBI	0.7785	0.1762	-0.3340 to 0.6864	25	6, 7
	TBI vs TBI+NTX	0.9773	0.06964	-0.4050 to 0.5443	25	7, 8
**IL-6 (8D)**	Sham vs TBI	0.0431*	-33.96	-67.11 to -0.8178	28	8, 8
	TBI vs TBI+NTX	0.0537	32.75	-0.3947 to 65.89	28	8, 8
**IL-6 (3M)**	Sham vs TBI	<0.0001****	-66.89	-100.3 to -33.52	27	8, 8
	TBI vs TBI+NTX	0.3394	20.83	-12.55 to 54.20	27	8, 8
**IL-12p70 (8D)**	Sham vs TBI	0.4049	34.41	-24.99 to 93.81	28	8, 8
	TBI vs TBI+NTX	0.2759	-40.06	-99.46 to 19.34	28	8, 8
**IL-12p70 (3M)**	Sham vs TBI	0.0002***	-105.8	-163.8 to -47.81	27	8, 8
	TBI vs TBI+NTX	0.0002***	105.0	47.02 to 163.0	27	8, 8
**IL-18 (8D)**	Sham vs TBI	0.3278	-3.516	-9.083 to 2.051	26	7, 8
	TBI vs TBI+NTX	0.0390*	5.600	0.2219 to 10.98	26	8, 8
**IL-18 (3M)**	Sham vs TBI	>0.9999	-0.1875	-8.872 to 8.497	28	8, 8
	TBI vs TBI+NTX	0.2600	-5.975	-14.66 to 2.710	28	8, 8
**IL-23 (8D)**	Sham vs TBI	0.2940	-253.9	-640.4 to 132.7	25	8, 8
	TBI vs TBI+NTX	0.0189*	510.3	69.49 to 951.0	25	8, 5
**IL-23 (3M)**	Sham vs TBI	0.7223	-163.8	-589.9 to 262.4	28	8, 8
	TBI vs TBI+NTX	0.9341	92.16	-334.0 to 518.3	28	8, 8
**TNFα (8D)**	Sham vs TBI	0.0012**	-115.4	-189.2 to -41.48	27	7, 8
	TBI vs TBI+NTX	0.0104*	89.00	17.64 to 160.4	27	8, 8
**TNFα (3M)**	Sham vs TBI	0.8478	34.59	-82.82 to 152.0	24	6, 8
	TBI vs TBI+NTX	0.9750	17.01	-95.51 to 129.5	24	8, 7
**CCL5 (8D)**	Sham vs TBI	<0.0001****	-5.425	-8.258 to -2.592	28	8, 8
	TBI vs TBI+NTX	0.0014**	4.325	1.492 to 7.158	28	8, 8
**CCL5 (3M)**	Sham vs TBI	0.7865	-1.000	-3.921 to 1.921	28	8, 8
	TBI vs TBI+NTX	0.4876	-1.538	-4.458 to 1.383	28	8, 8
**IL-4 (8D)**	Sham vs TBI	0.0239*	2.668	0.2864 to 5.049	26	8, 7
	TBI vs TBI+NTX	0.0095**	-3.005	-5.387 to -0.6239	26	7, 8
**IL-4 (3M)**	Sham vs TBI	0.8744	0.6036	-1.614 to 2.821	23	7, 8
	TBI vs TBI+NTX	0.9406	0.4750	-1.839 to 2.789	23	8, 6

Multiplex custom cytokine array on serum samples. A total of 32 analytes, including proinflammatory and anti-inflammatory cytokines and chemokines, and growth factors were investigated during the acute and chronic phases of injury. *p<0.05, **p<0.01, ***p<0.001, ****p<0.0001 with respect to sham vs TBI, and TBI vs TBI/NTX. One-way ANOVA with Tukey's post-hoc analysis. Abbreviations: TBI, traumatic brain injury; NTX, naltrexone. For methods, see Supporting Information 5.

Supporting Table 3. Quantification of NeuN positive cells in the neocortex

	Treatment Groups	p-value	Mean difference	95% CI of difference	DF	Sample size
**8 days**	Sham vs TBI	0.0150*	526.9	88.73 to 965.0	20	6, 6
	TBI vs TBI+NTX	0.0165*	-520.1	-958.2 to -81.98	20	6, 6
**3 months**	Sham vs TBI	0.9913	39.94	-344.0 to 423.9	21	6, 6
	TBI vs TBI+NTX	0.9772	53.55	-316.4 to 423.5	21	6, 7

Comparison between NeuN (neuronal markers) positive cells between the groups at 8 days and 3 months post-TBI. A significant neuronal loss was observed in the regions with high FJB positive cells in the cortex. NTX prevented the cell loss. *$p<0.05$ for sham vs TBI, and TBI vs TBI+NTX. One-way ANOVA with Tukey's post-hoc analysis.

Supporting Table 4. Fractional anisotropy analyses from DTI-MRI

Region	OB		GP		HYP		CBL		FIM		LV		3V		AC		MBU	
**Groups**	**Pre**	**Post**	**Pre**	**Post**	**Pre**	**Post**	**Pre**	**Post**	**Pre**	**Post**	**Pre**	**Post**	**Pre**	**Post**	**Pre**	**Post**	**Pre**	**Post**
**TBI (8 d)**	0.259	0.233*	0.409	0.382*	0.342	0.322*	0.291	0.265*	0.482	0.440*	0.314	0.263*	0.234	0.222*	0.368	0.331*	0.352	0.342*
**TBI+NTX (8 d)**	0.268	0.265	0.449	0.453	0.323	0.331	0.302	0.304	0.445	0.431	0.280	0.290	0.228	0.236	0.358	0.363	0.367	0.354
**TBI (3 M)**	0.277	0.245*	0.365	0.330	0.338	0.295	0.269	0.242*	0.481	0.398*	0.338	0.313*	0.240	0.237	0.362	0.350*	0.339	0.300*
**TBI+NTX (3 M)**	0.268	0.243	0.449	0.388*	0.323	0.278*	0.302	0.253*	0.445	0.385*	0.280	0.236	0.228	0.213	0.358	0.318	0.367	0.320

Supporting Table 4 continued. Fractional anisotropy analyses from DTI-MRI

Region	FX		4V		HBU		SPC		PG		AMY		MED		PON	
**Groups**	**Pre**	**Post**	**Pre**	**Post**	**Pre**	**Post**	**Pre**	**Post**	**Pre**	**Post**	**Pre**	**Post**	**Pre**	**Post**	**Pre**	**Post**
**TBI (8 d)**	0.342	0.331*	0.282	0.271*	0.343	0.365	0.139	0.123*	0.265	0.234	0.439	0.439*	0.333	0.319*	0.359	0.332*
**TBI+NTX (8 d)**	0.316	0.316	0.276	0.285	0.393	0.380	0.024	0.025	0.266	0.256	0.448	0.427	0.363	0.360	0.408	0.375
**TBI (3 M)**	0.347	0.299*	0.282	0.231*	0.314	0.259*	0.254	0.229*	0.284	0.292	0.410	0.392	0.334	0.291*	0.371	0.333*
**TBI+NTX (3 M)**	0.316	0.276	0.276	0.227*	0.393	0.331*	0.024	0.029	0.266	0.226	0.448	0.383*	0.363	0.316	0.408	0.342

Mean FA values of different brain regions between TBI and TBI+NTX groups at 8 days and 3 months. All the post-injury values were normalized against the pre-injury group. Therefore, the higher the value, the higher is the FA, whereas lower values represent decreased FA. The decrease in FA was higher in TBI at both 8 days and 3 months, whereas lesser or no changes in FA were observed in the TBI+NTX group, compared to the pre-injury group. An Asterisk represents a decrease in FA after injury. Abbreviations: Olfactory Bulb (OB), Globus Pallidus (GP), Hypothalamus (HYP), Cerebellum (CBL), Fimbria (FIM), Lateral Ventricles (LV), 3rd Ventricle (3V), Anterior Commissure (AC), Midbrain Unsegmented (MBU), Fornix (FX), 4th Ventricle (4V), Hind Brain Unsegmented (HBU), Spinal Cord (SPC), Pineal Gland (PG), Amygdala (AMY), Medulla (MED), Pons (PON)

Supporting Table 5. Spontaneous seizure shown by individual mouse

ID (TBI)	S1	S2	S3	S4	S20	S21	S23	
SRS	0	1	0	11	5	3	6	
		Stage 5: 1		Stage 3: 1<br>Stage 4: 1<br>Stage 5: 9	Stage 2: 5	Stage 2: 3	Stage 2: 6	
ID (T+N)	S6	S7	S8	S9	S24	S25	S26	S27
SRS	0	0	0	0	0	0	0	0

Spontaneous seizures shown by each mouse during three months of observation period from TBI (top panel) and TBI+NTX group (lower panel). For example, for mouse S4 (top panel) showed 11 seizures after TBI, out of which one was stage 3 seizure, one stage 4 and nine were stage 5 seizures. None of the mice showed any seizure when TBI mice were treated with NTX (bottom panel). Abbreviations: SRS, spontaneous recurrent seizures; T+N, traumatic brain injury + naltrexone. Weekly seizures and duration for each SRS is shown in figure 7.

Supporting Figure 1. Standardization of the Experimental Design

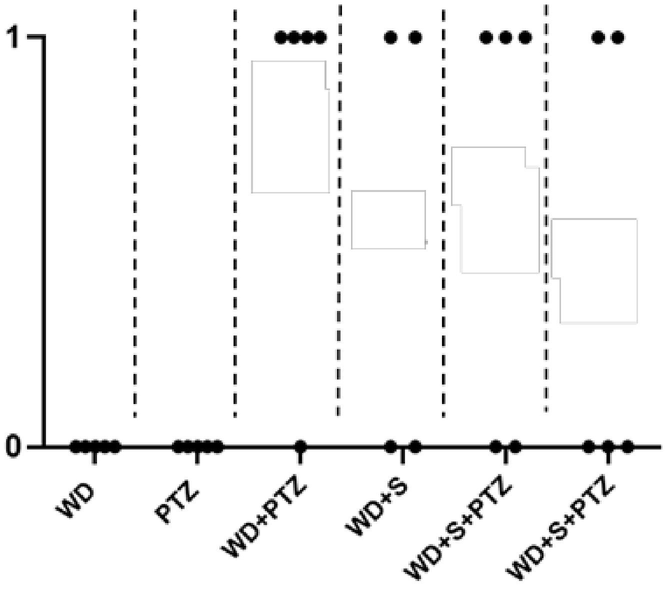

Group →	WD (Grp I)	PTZ (Grp II)	WD+PTZ (Grp III)	WD+S (Grp IV)	WD+S+PTZ Grp (V)	WD+S+PTZ (Grp VI)
Number of Animals	0	0	0	1	1	0
	0	0	1	0	0	0
	0	0	1	0	1	1
	0	0	1	1	0	0
	0	0	1	-	1	1

Pilot experiments were performed in six different groups (n=4-5) to check mortality, prior to the main experiment. Traumatic brain injury induced by WD (Grp I) or PTZ (Grp II) on its own did not cause any mortality, whereas

PTZ, when administered on the same day of WD injury, caused 80% mortality (Grp III). In group IV, about half of the animals died, when surgery was performed after WD on the same day. In group V and VI, TBI was induced on day 1, electrodes were implanted on day 2 and the PTZ test was performed on day 3 and 4. 3 out 5 animals in group V and 2 out 5 animals on day 4 died after the PTZ test. These observations from the pilot studies, and other supporting data, were used to design the main experiment shown in supporting figure 2. Abbreviations: TBI, traumatic brain injury; WD, weight drop; PTZ, pentylenetetrazol.

Supporting Figure 2. Neurobehavioral status of the mice after TBI

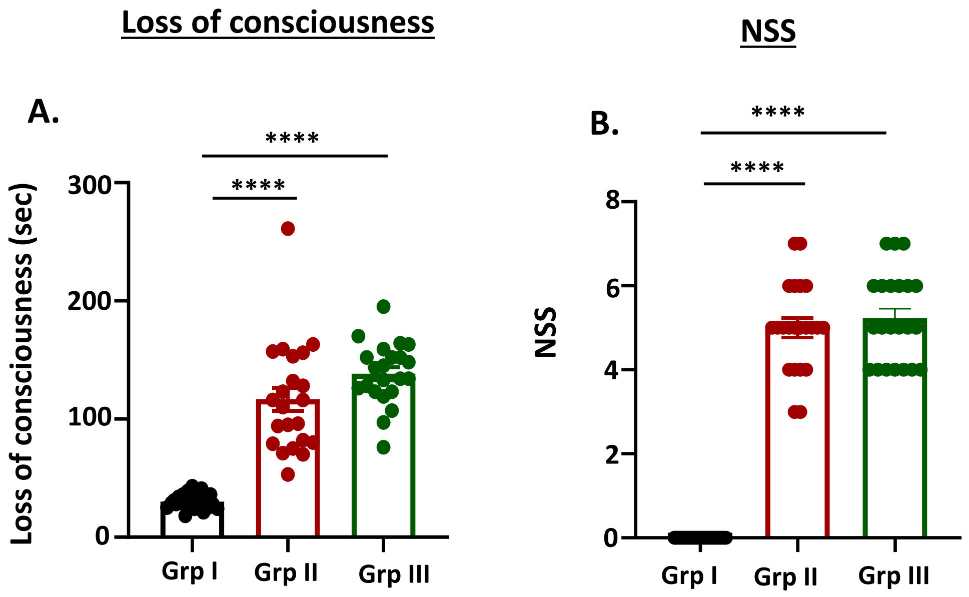

Functional outcome after TBI. The severity of TBI was determined by two factors, ie., LOC and NSS. A) LOC is the time taken by the animal to regain consciousness after TBI. LOC was significantly higher in all the mice that had TBI (Grp II and Grp III), when compared to sham (Grp I). Animals in Grp II and Grp III, after TBI, were randomly selected for the TBI and TBI+NTX groups. The NSS test consists of assessments of: loss of reflexes (forelimb and hindlimb, pinna, cornea, eye blink and startle reflex tests), inability to walk (hemiplegia, monoparesis, hemiparesis), inability to exit, loss of seeking behavior, prostration, whisking and tremors. If a loss or impairment in any of these behaviors was observed, then a value of 1 was assigned to the animal (Fujimoto et al., 2004; Chen et al., 1996; Flierl et al., 2009; Hamm, 2001; Gold et al., 2013, Hunter et AL., 2000). Therefore, higher values indicate severe neurological impairment. The experiment was repeated twice with each animal ending up with a total of three injuries (one injury/hour). ****$p < 0.0001$, One-way ANOVA with Tukey's post-hoc test; $n = 22$. All the data is represented as SEM. Abbreviations: TBI, traumatic brain injury; LOC, loss of consciousness; NSS, neurological severity score.

Full Unedited gel for Figure 1A. Anti-Phospho-mu Opioid Receptor (S375) Antibody (different contrast intensities)

8-Day study

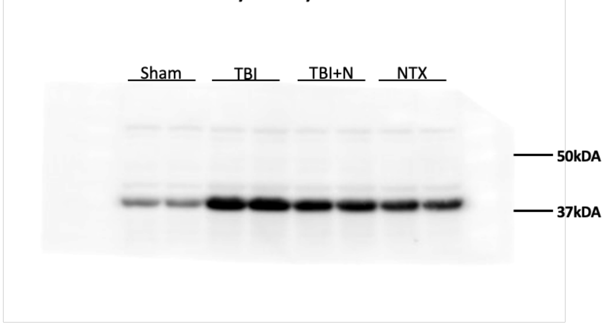

Actin

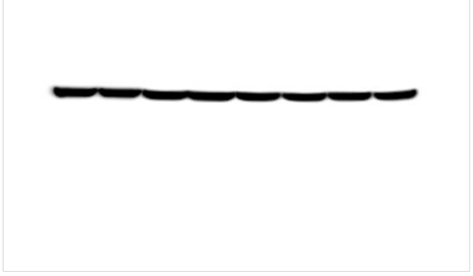

3M-Month study

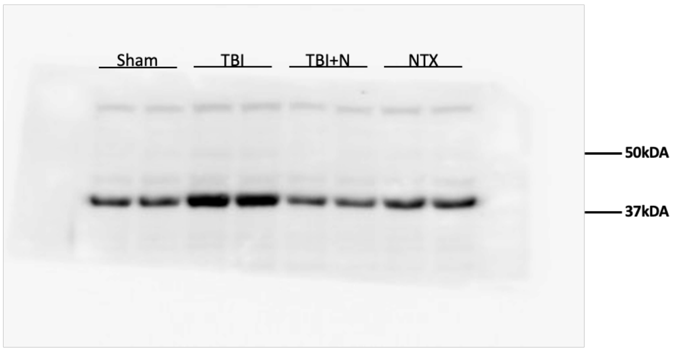

Actin

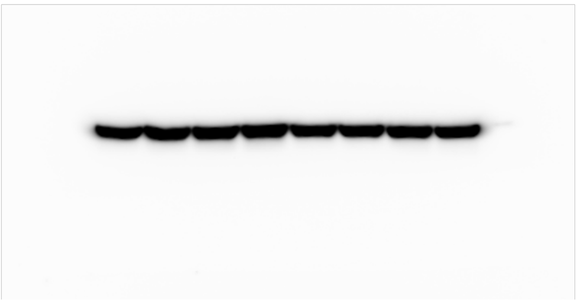

8-Day study

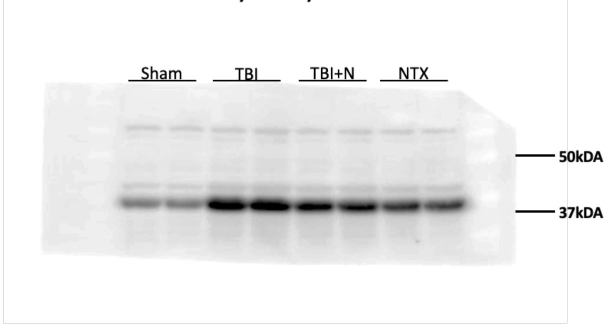

Actin

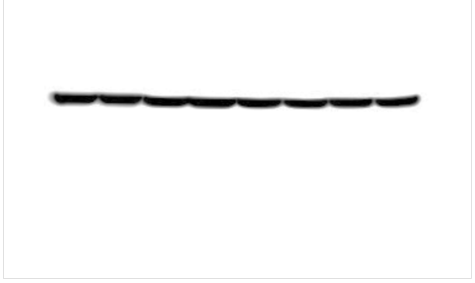

3M-Month study

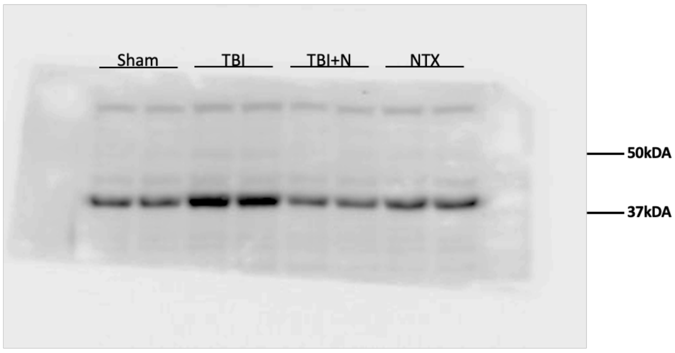

Actin

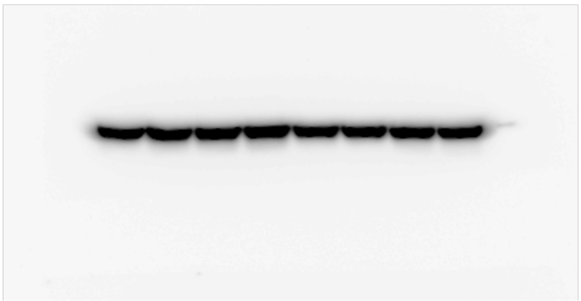

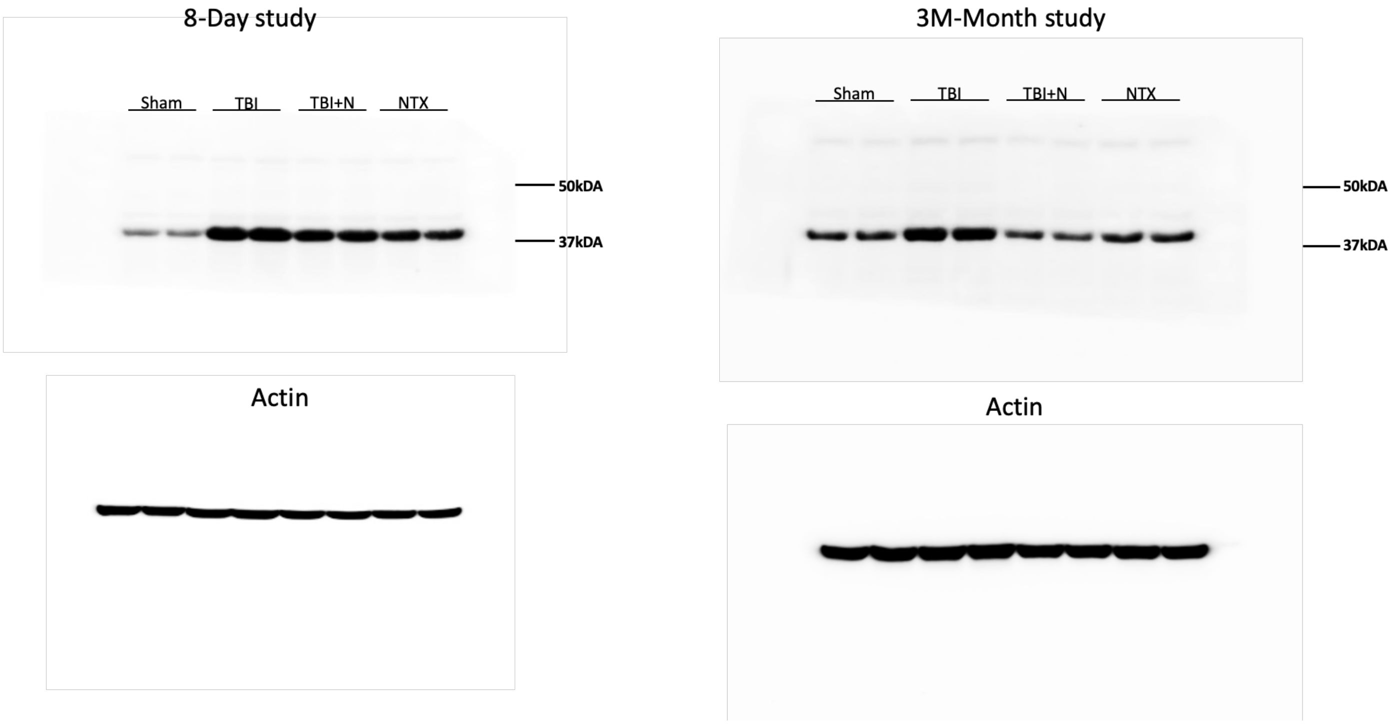

Full Unedited gel for Figure 1B. Anti-Mu Opioid Receptor N-terminal [NT2] Antibody (different contrast intensities)

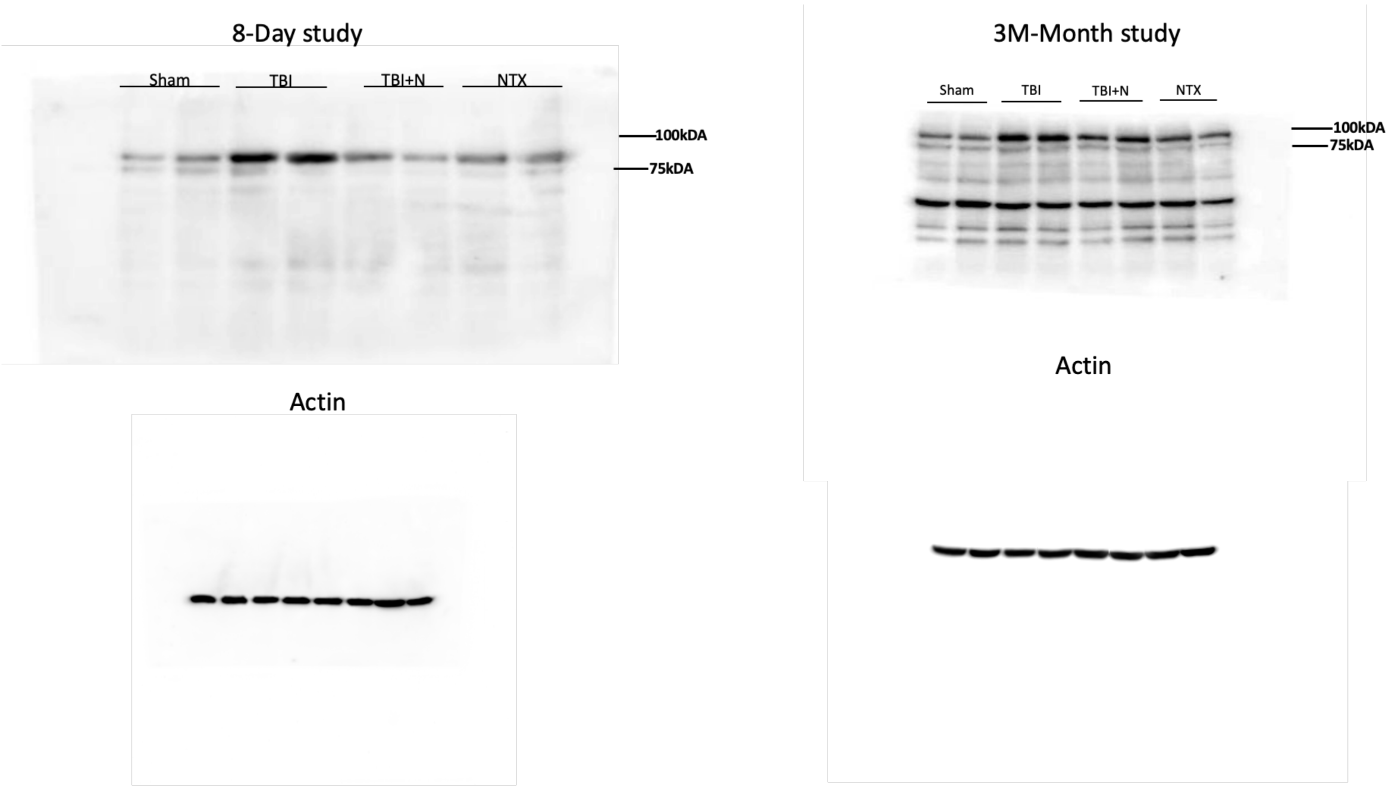

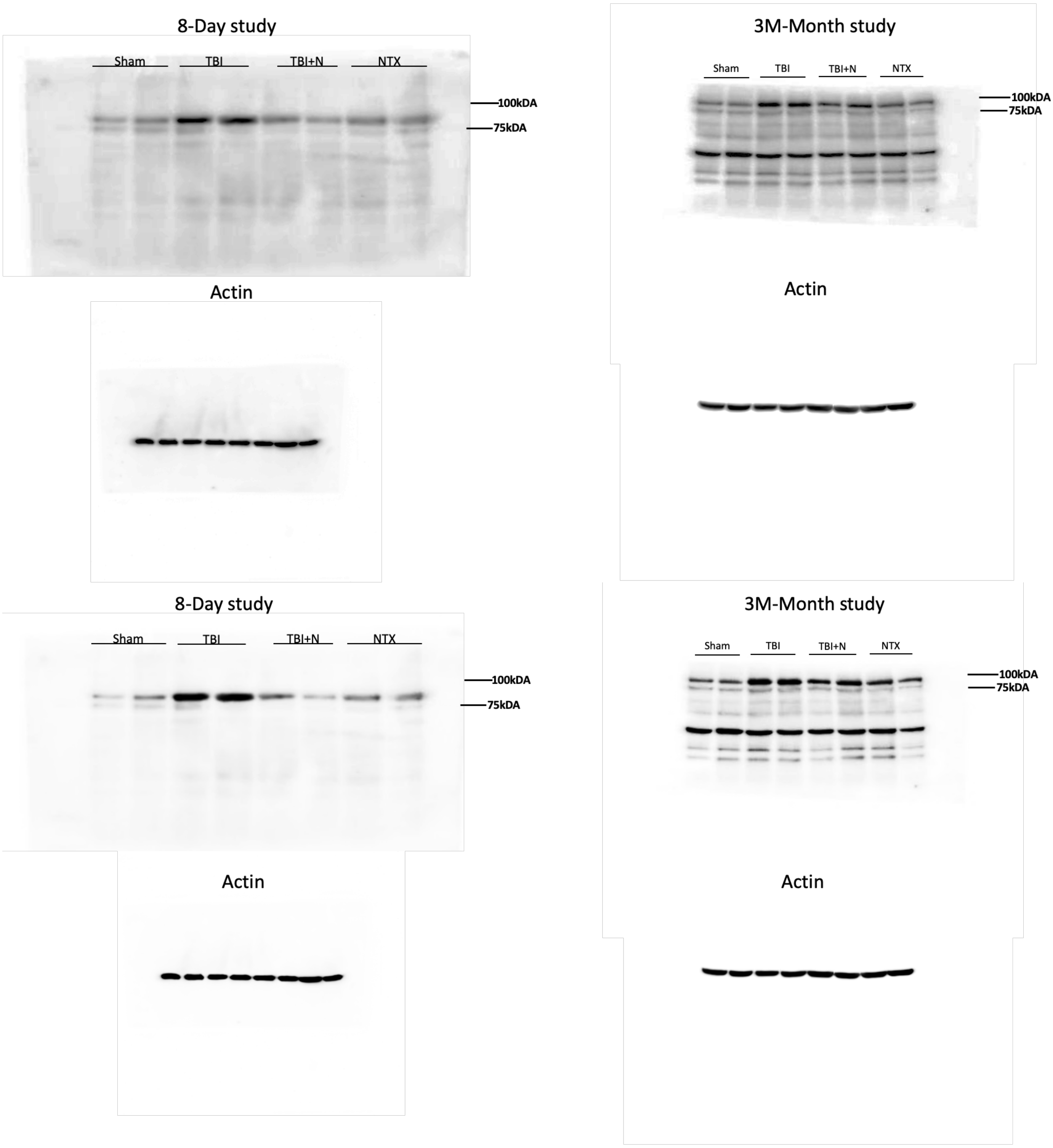

Full Unedited gel for Figure 2B. Anti-Phosphor-p38 MAPK Antibody (different contrast intensities)

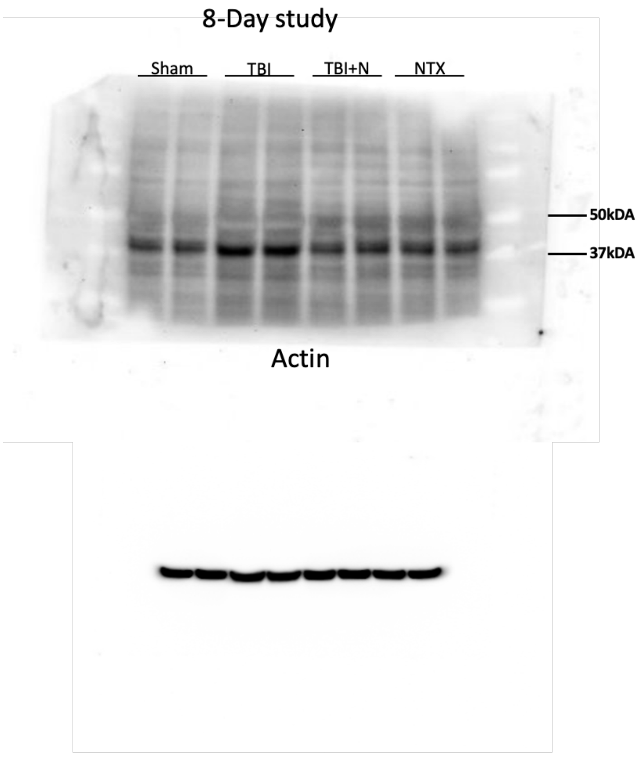
8-Day study
Sham
TBI
TBI+N
NTX
50kDA
37kDA
Actin

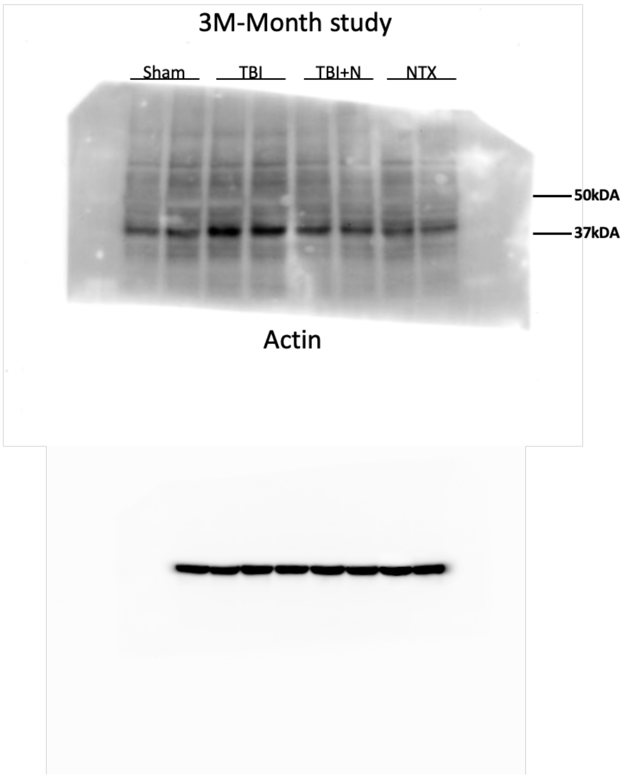
3M-Month study
Sham
TBI
TBI+N
NTX
50kDA
37kDA
Actin

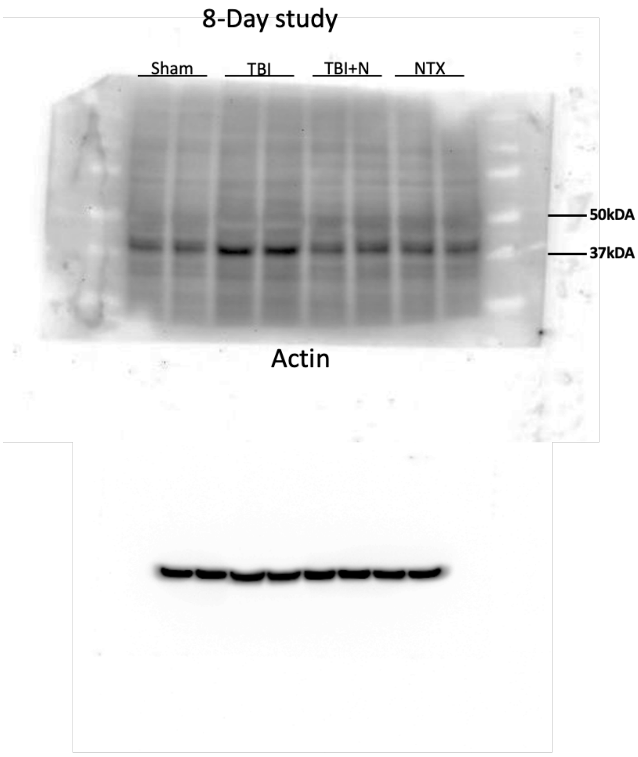
8-Day study
Sham
TBI
TBI+N
NTX
50kDA
37kDA
Actin

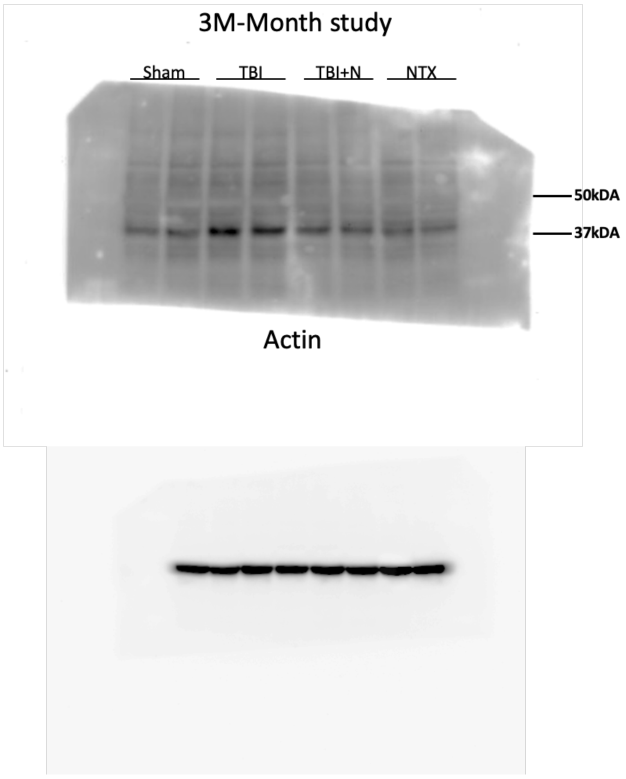
3M-Month study
Sham
TBI
TBI+N
NTX
50kDA
37kDA
Actin

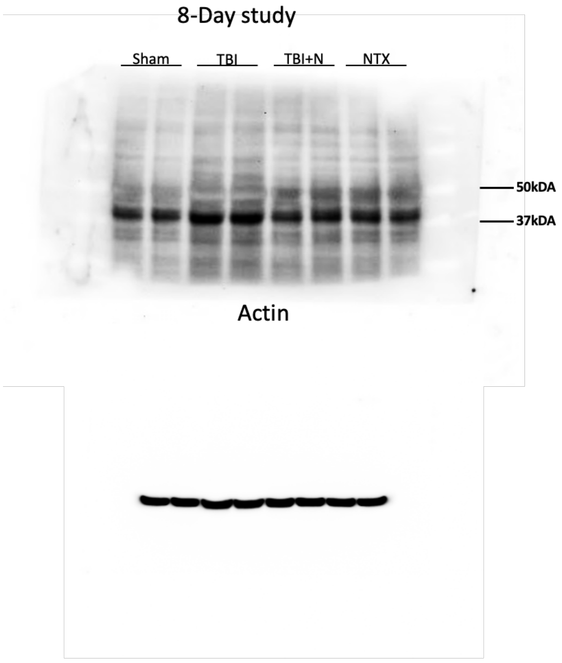

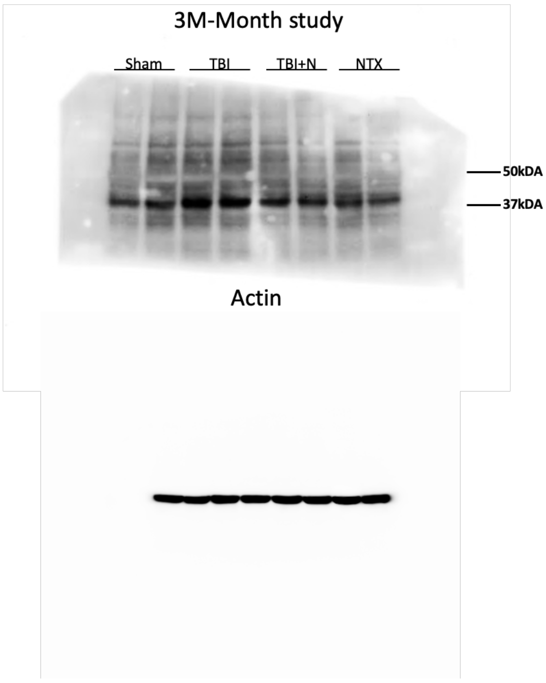

Full Unedited gel for Figure 2C. Anti 3-NT Antibody (different contrast intensities)

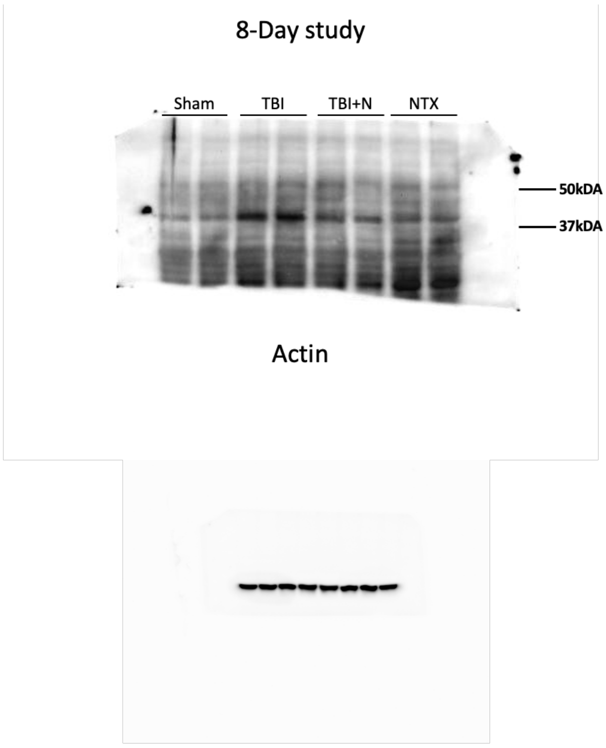

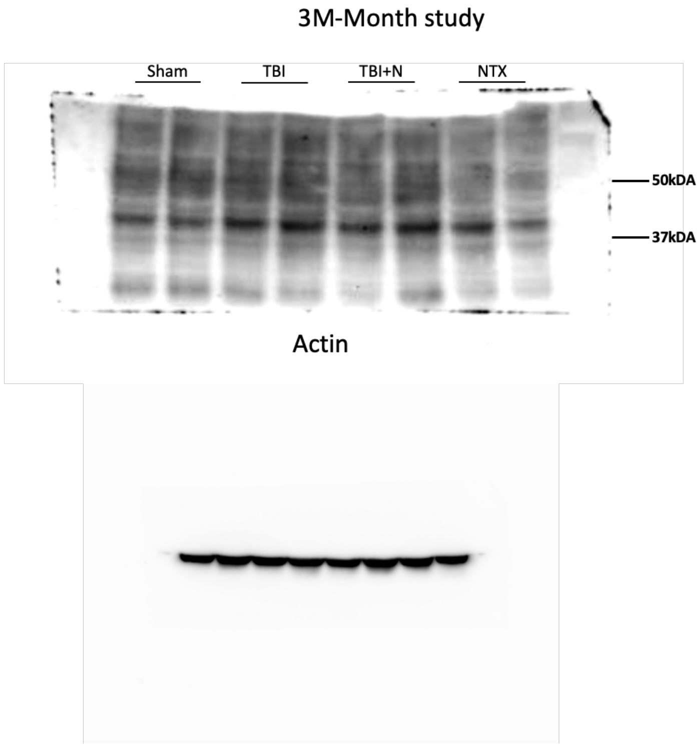

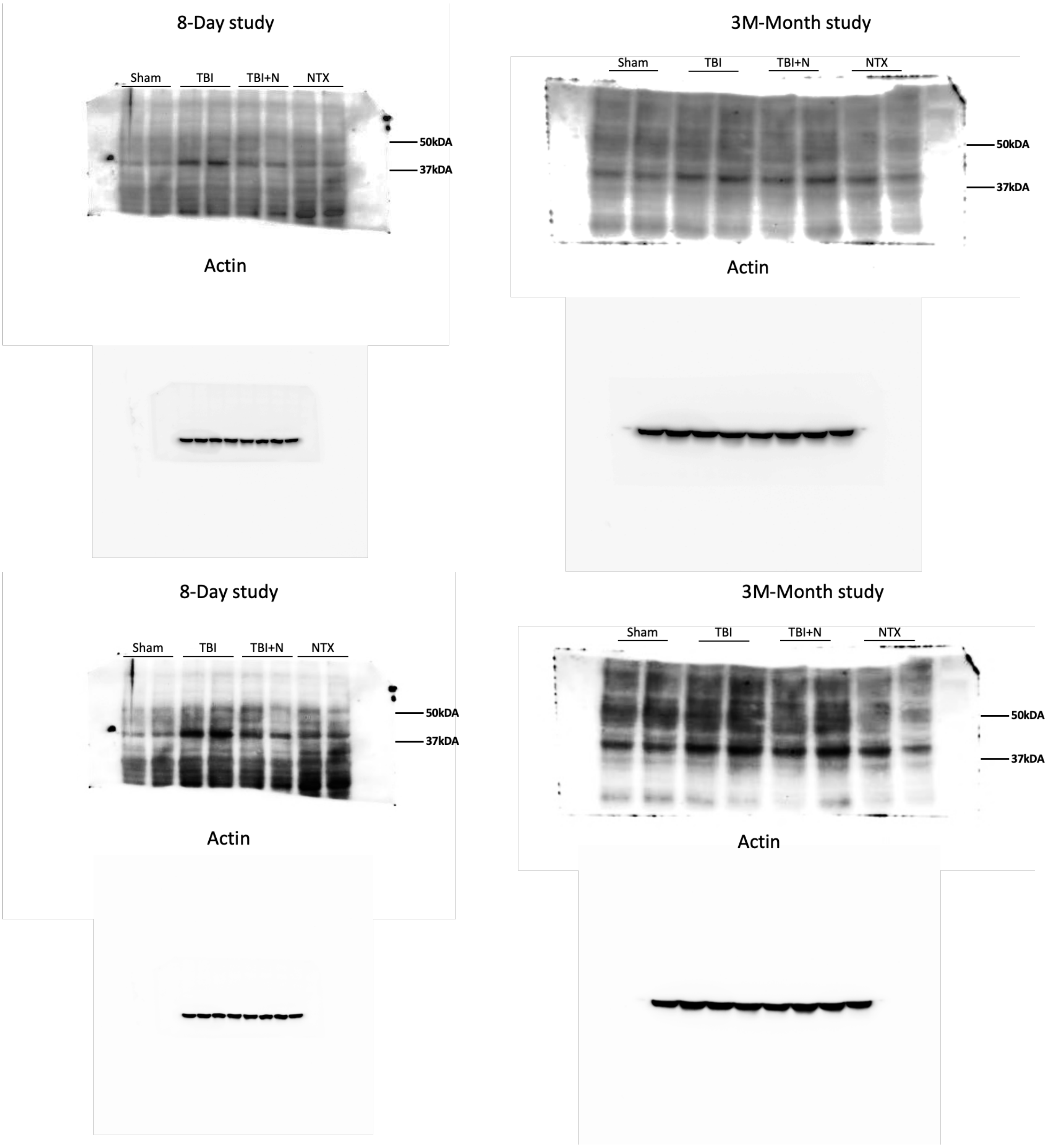

Full Unedited gel for Figure 2D. Anti iNOS Antibody (different contrast intensities)

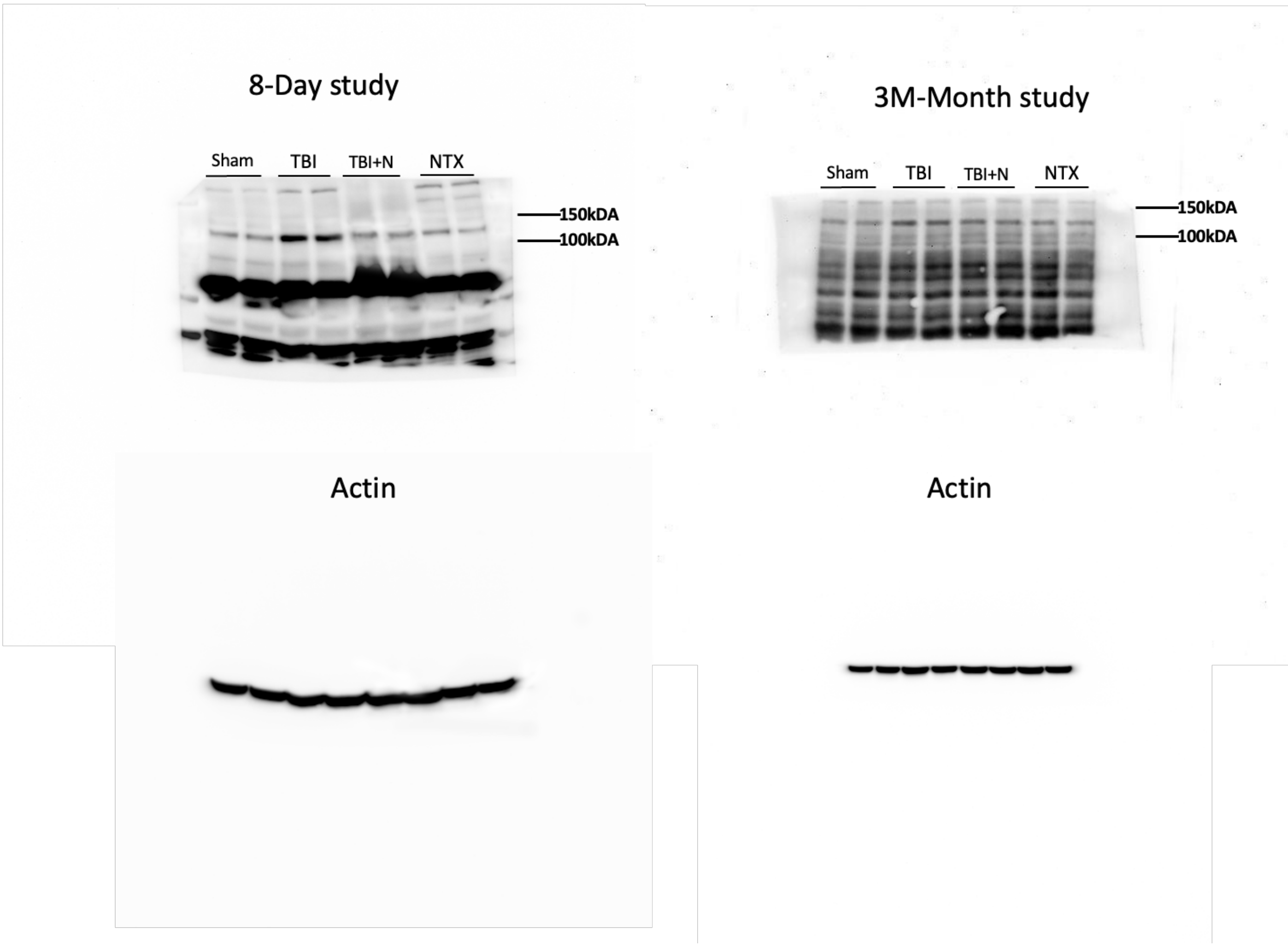
8-Day study
Sham
TBI
TBI+N
NTX
150kDA
100kDA
Actin
3M-Month study
Sham
TBI
TBI+N
NTX
150kDA
100kDA
Actin

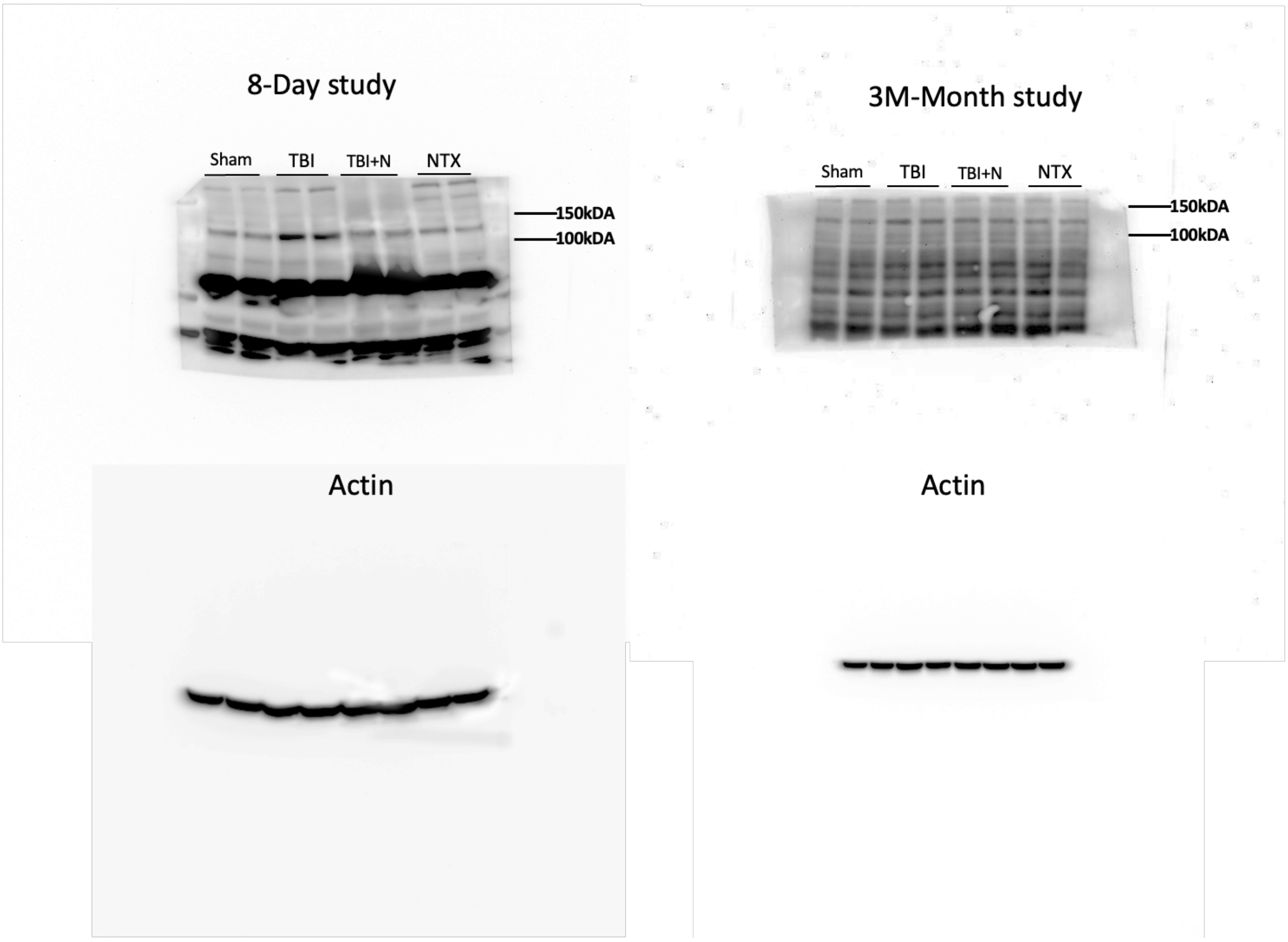
8-Day study
Sham
TBI
TBI+N
NTX
150kDA
100kDA
Actin
3M-Month study
Sham
TBI
TBI+N
NTX
150kDA
100kDA
Actin

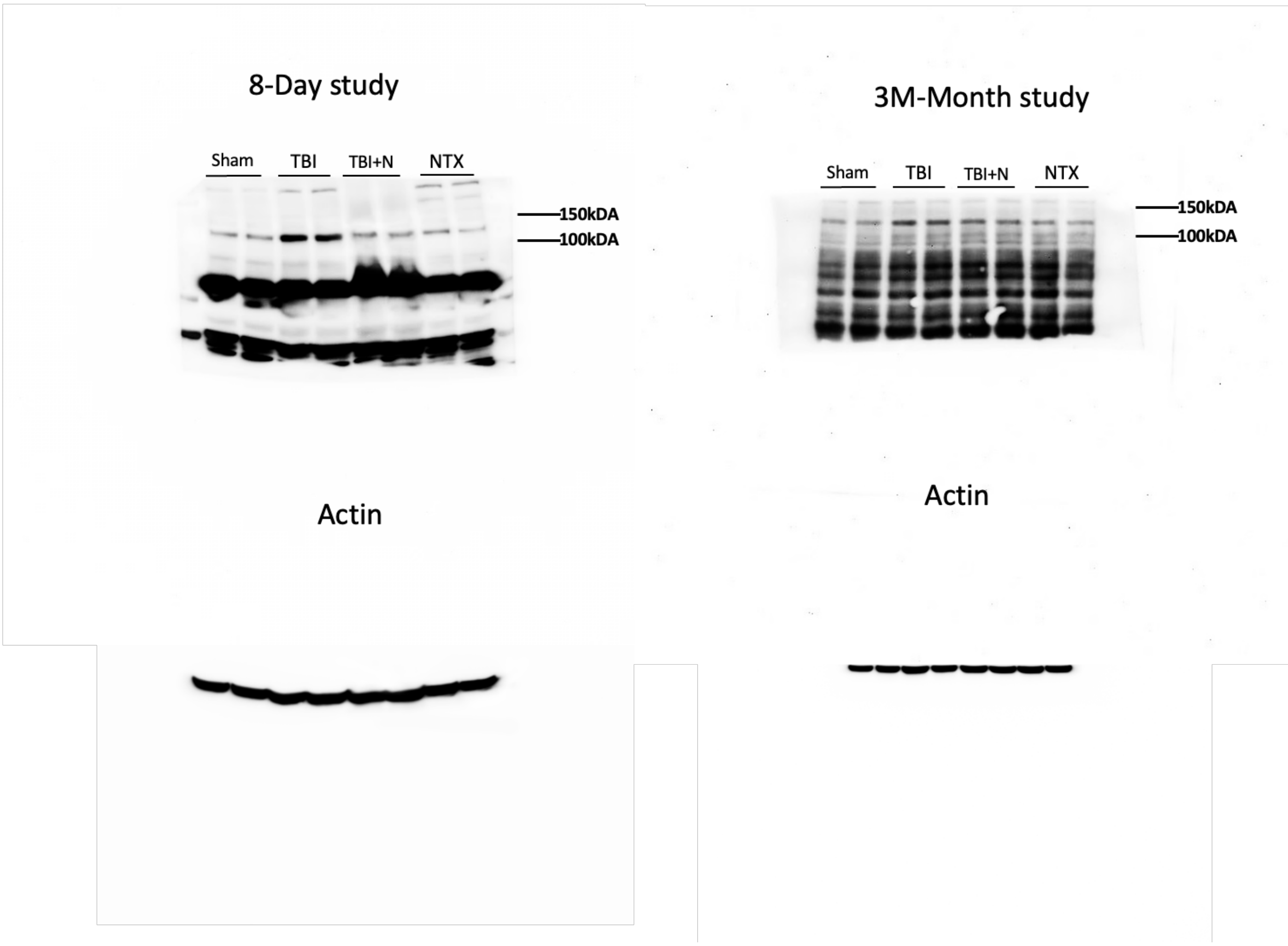
8-Day study
Sham
TBI
TBI+N
NTX
150kDA
100kDA
Actin
3M-Month study
Sham
TBI
TBI+N
NTX
150kDA
100kDA
Actin
